# Molecular Editing Reveals a Bilobalide Chemotype that Attenuates Prolyl Endopeptidase (PREP)‐Linked Neuroinflammation

**DOI:** 10.1002/advs.202600011

**Published:** 2026-08-11

**Authors:** Chanin Sillapachaiyaporn, Wenjing Wang, Yao Qin, Lili Yan, Stephan Scheeff, Xu He, Qiong Wu, Yue Lin, Jinsai Shang, Xin Ye, Jing Liu, Kailei Sun, Chun Wai Yeung, Junzhe Huang, Zhenghui Chen, Xiaodong Liu, Yiqun Geng, Shannon Wing Ngor Au, Bonaventure Y. Ip, Ho Ko, Billy Wai‐Lung Ng

**Affiliations:** ^1^ Guangdong‐Hong Kong‐Macao Joint Laboratory for New Drug Screening School of Pharmacy Faculty of Medicine The Chinese University of Hong Kong Hong Kong SAR China; ^2^ Li Ka Shing Institute of Health Sciences Faculty of Medicine The Chinese University of Hong Kong Hong Kong SAR China; ^3^ School of Basic Medical Sciences Guangzhou National Laboratory State Key Laboratory of Respiratory Disease Guangzhou Medical University Guangzhou China; ^4^ Cipher Gene Ltd Chengdu China; ^5^ Center for Protein Science and Crystallography School of Life Sciences The Chinese University of Hong Kong Hong Kong China; ^6^ Department of Medicine and Therapeutics Faculty of Medicine The Chinese University of Hong Kong Hong Kong SAR China; ^7^ State Key Laboratory of Bioactive Substance and Function of Natural Medicines Beijing Key Laboratory of Active Substances Discovery and Druggability Evaluation Institute of Materia Medica Chinese Academy of Medical Sciences and Peking Union Medical College Beijing China; ^8^ Department of Anesthesia and Intensive Care Faculty of Medicine The Chinese University of Hong Kong Hong Kong SAR China; ^9^ Peter Hung Pain Research Institute Faculty of Medicine The Chinese University of Hong Kong New Territories Hong Kong SAR China; ^10^ Gerald Choa Neuroscience Institute The Chinese University of Hong Kong Hong Kong SAR China

**Keywords:** bilobalide, natural product modification, neuroinflammation, PREP, prolyl endopeptidase, target identification

## Abstract

Natural products offer privileged three‐dimensional chemotypes for drug discovery, yet scaffold lability and limited analogue space often impede mechanism‐guided optimization. Herein, we show that molecular editing can reveal a natural product‐derived chemotype that attenuates neuroinflammation via prolyl endopeptidase (PREP) inhibition. **BB10**, a chemically stabilized bilobalide analogue generated via lactone‐to‐lactam molecular editing, significantly suppressed LPS‐induced microglial activation and reduced levels of key inflammatory mediators. Unbiased cellular target identification (CETSA‐MS) identified PREP as the top thermally stabilized target, supporting cellular target engagement. **BB10** inhibited PREP activity in both cellular and recombinant enzyme assays, implicating PREP inhibition as a mechanism contributing to its anti‐neuroinflammatory activity. Guided by this target‐based insight, structural optimization afforded a fluorinated analogue, **BB56**, with improved PREP inhibition and superior anti‐inflammatory efficacy. Mechanistically, **BB56** attenuated p38/NF‐κB signaling and reduced NLRP3 upregulation, and PREP knockdown abrogated the anti‐inflammatory effects. Finally, **BB56** provided functional rescue in a zebrafish model of neuroinflammation. Collectively, this work establishes a molecular editing‐enabled approach to a natural product chemotype that modulates neuroinflammation through PREP inhibition.

## Graphical abstract

Molecular editing of bilobalide uncovers **BB56**, a PREP‐engaging chemotype with enhanced anti‐inflammatory activity in microglia. Integrated CETSA‐MS, SAR, and pathway analyses link BB56 to attenuation of p38 MAPK/NF‐κB and NLRP3‐associated inflammatory signaling, highlighting bilobalide editing as a strategy for developing neuroinflammation‐modulating natural product analogues.

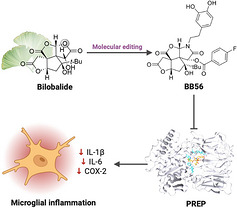

## 1 Introduction

Natural products are a privileged source of biologically rich, conformationally defined chemotypes for drug discovery, but their translation into mechanism‐guided chemical biology is frequently limited by scaffold lability, restricted analogue space, and incomplete target annotation in cells [1]. Molecular editing offers a practical strategy to convert fragile natural product scaffolds into tractable, target‐engaged drug‐like molecules using simple yet powerful chemical transformations [2].

The natural product bilobalide, a highly oxygenated sesquiterpene trilactone from *Ginkgo biloba*, has long been recognized for its neuroprotective and anti‐inflammatory properties [3, 4, 5, 6], supported by favorable oral bioavailability and notable blood‐brain barrier penetration [7, 8]. Nevertheless, the intrinsic structural complexity and chemical lability of its trilactone core have posed major challenges for systematic derivatization, target identification, and mechanistic investigation [9, 10]. In our previous works, we addressed these limitations by developing lactone‐to‐lactam molecular editing strategies that confers enhanced chemical stability and synthetic tractability [11, 78]. Specifically, lactamization of the D‐ring is proposed to stabilize the adjacent C‐ring lactone, thereby mitigating degradation liabilities inherent to the parent scaffold. In addition, this late‐stage modification enables streamlined access to a diversified bilobalide‐derived chemical space while modulating key pharmacological properties, thus providing a versatile platform for structure‐activity relationship exploration and target discovery.

Neuroinflammation is a defining feature of many neurological diseases, including stroke [12, 13, 14] and Alzheimer's disease [15, 16, 17]. It is primarily driven by dysregulated activation of microglia, the innate immune cells of the central nervous system [18, 19]. Dysregulated microglial activation triggers excessive release of cytokines and reactive oxygen species (ROS) [20, 21], creating a neurotoxic environment that accelerates neuronal injury and disease progression [22, 23, 24]. Persistent microglial overactivation is consistently observed in both animal models and patient tissues, underscoring its pathogenic relevance and positioning microglial modulation as an attractive therapeutic strategy [25, 26]. Here, we asked whether bilobalide‐derived lactam analogues, with altered pharmacological properties, can advance beyond phenotypic anti‐inflammatory activity to achieve mechanism‐defined modulation of neuroinflammation.

Using unbiased target identification via cellular thermal shift assay‐mass spectrometry (CETSA‐MS), we herein identified PREP as a functionally relevant cellular target engaged by the bilobalide‐derived analogue **BB10**. PREP (also known as prolyl oligopeptidase) is a serine protease that selectively cleaves proline‐containing peptides and has emerged as a promising therapeutic target in neuroinflammatory conditions [27, 28]. PREP is abundantly expressed in the brain and modulates the activity of neuropeptides such as substance P and α‐melanocyte‐stimulating hormone [29], both of which are linked to inflammatory signaling [30, 31, 32, 33, 34]. Elevated PREP activity has been observed under neuroinflammatory conditions, promoting cytokine production, oxidative stress, and glial activation [35]. Conversely, pharmacological inhibition of PREP suppresses inflammation‐associated signaling pathways, including p38 MAPK and NF‐κB [36], attenuates microglial and astrocytic activation, suppresses neurotoxic mediator release, and enhances anti‐inflammatory responses, ultimately protecting neurons from inflammatory damage [27].

Building on this target identification, we further developed an optimized analogue, **BB56**, which effectively attenuates lipopolysaccharide (LPS)‐induced microglial activation and suppresses PREP‐linked inflammatory signaling. Together, these findings establish a PREP‐targeting chemotype derived from a natural product and deliver a mechanistically supported chemotype for microglial neuroinflammatory modulation.

## 2 Results and Discussion

### 2.1 BB10 Inhibits Neuroinflammation in LPS‐induced BV‐2 Microglial Cells

**FIGURE 1:**
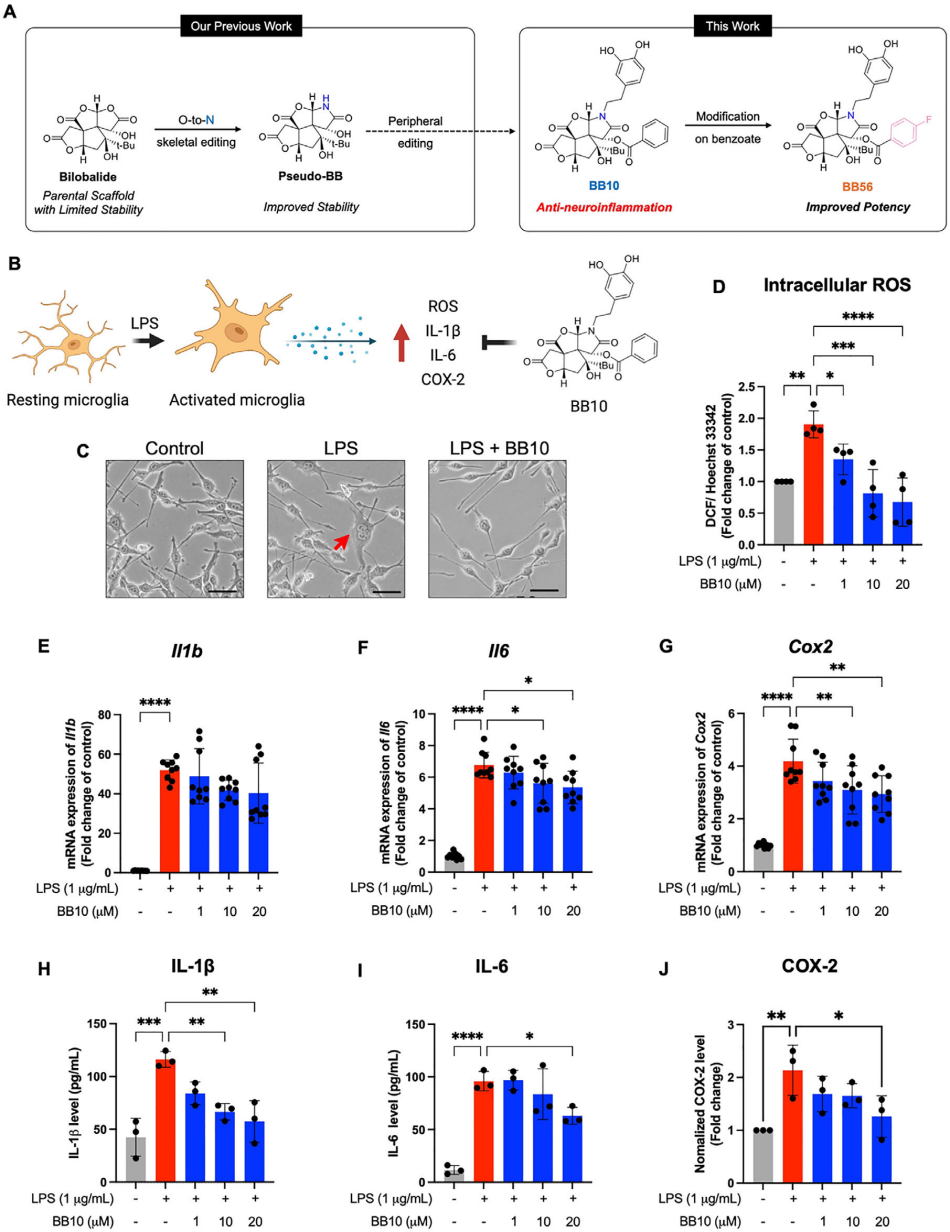
BB10 attenuates neuroinflammation in LPS‐stimulated BV‐2 microglial cells. (A) Conceptual overview of molecular editing of bilobalide. (B) Schematic illustration showing the proof‐of‐concept of anti‐neuroinflammatory activity of **BB10**. (C) Representative microscopic images showing morphological changes of BV‐2 microglia after LPS stimulation for 24 h, with or without **BB10** (20 µm). Red arrows indicate activated microglia. 20X magnification, scale bar = 50 µm. (D) Intracellular reactive oxygen species (ROS) levels were measured in LPS‐stimulated BV‐2 cells co‐treated with **BB10** (1, 10, and 20 µm) for 24 h. **BB10** attenuated LPS‐induced ROS production in BV‐2 cells. DCF (dichlorodihydrofluorescein) indicates ROS level. (E–G) **BB10** suppressed the mRNA expression of pro‐inflammatory genes *Il1b*, *Il6*, and *Cox2* in LPS‐stimulated BV‐2 cells. Cells were co‐treated with LPS and **BB10** for 6 h, followed by RT‐qPCR analysis. (H,I) **BB10** reduced IL‐1β and IL‐6 cytokine secretion in LPS‐activated BV‐2 cells after 24 h co‐treatment, as measured by ELISA. (J) Western blot analysis showing that **BB10** decreased COX‐2 protein expression in LPS‐stimulated BV‐2 cells after 6 h treatment (refer to original immunoblots in Figure S11). Data are presented as mean ± SD (n = 4 for panel D; n = 9 for panels E‐G; and n = 3 for panels H‐J). Statistical analysis was performed using one‐way ANOVA followed by Dunnett's post hoc test. ^*^ *p* < 0.05, ^**^ *p* < 0.01, ^***^ *p* < 0.001, ^****^ *p* < 0.0001.

Using a simple yet powerful lactone‐to‐lactam molecular editing strategy, we previously generated a chemically stabilized, synthetically accessible bilobalide lactam scaffold and identified **BB10** as a hit compound with moderate anti‐ferroptotic activity [11]. We herein asked whether scaffold editing could unlock additional pharmacological activities beyond ferroptosis. Accordingly, we profiled **BB10** across complementary cellular readouts relevant to neuroinflammation to uncover latent bioactivity and define molecular target(s) (Figure 1A).

In neuroinflammation, microglia are overactivated by pathogenic aggregates such as amyloid‐β and α‐synuclein, which signal through toll‐like receptor 4 (TLR4) to initiate a pro‐inflammatory cascade that drives neuronal injury [17, 37, 38, 39, 40]. To model this clinically relevant pathway, we employed the LPS‐induced microglial activation system, as LPS is a potent TLR4 agonist that reliably engages the same canonical signaling axis and provides a robust platform for dissecting microglial inflammatory responses [41, 42]. Upon activation, microglia adopt an amoeboid morphology and upregulate the expression of pro‐inflammatory mediators, including IL‐1β, IL‐6, COX‐2, and ROS that drive neuroinflammation [18, 43, 44]. To determine whether **BB10** can also modulate microglial inflammatory activation, we examined its effects in LPS‐stimulated BV‐2 microglia (Figure 1B).

Morphological assessment revealed that LPS markedly induced microglial activation, characterized by a transition from a resting, spindle‐like morphology to an amoeboid form (Figure 1C). Treatment with **BB10** attenuated these morphological changes (Figure 1C). Consistent with this morphological protection, **BB10** significantly reduced intracellular ROS levels in LPS‐stimulated BV‐2 cells (Figure 1D). Quantitative RT‐PCR analysis showed that LPS markedly upregulated *Il1b*, *Il6*, and *Cox2* transcripts, whereas **BB10** treatment suppressed their expression. **BB10** (10 and 20 µm) significantly reduced *Il6* and *Cox2* levels (Figure 1E–G). Correspondingly, **BB10** treatment decreased IL‐1β and IL‐6 secretion (Figure 1H,I) and reduced COX‐2 protein expression (Figure 1J). Taken together, these findings demonstrate that **BB10** suppresses LPS‐induced microglial activation and inflammation by inhibiting oxidative stress and suppressing key pro‐inflammatory genes and proteins.

### 2.2 BB10 Engages PREP and Inhibits PREP Enzymatic Activity

**FIGURE 2:**
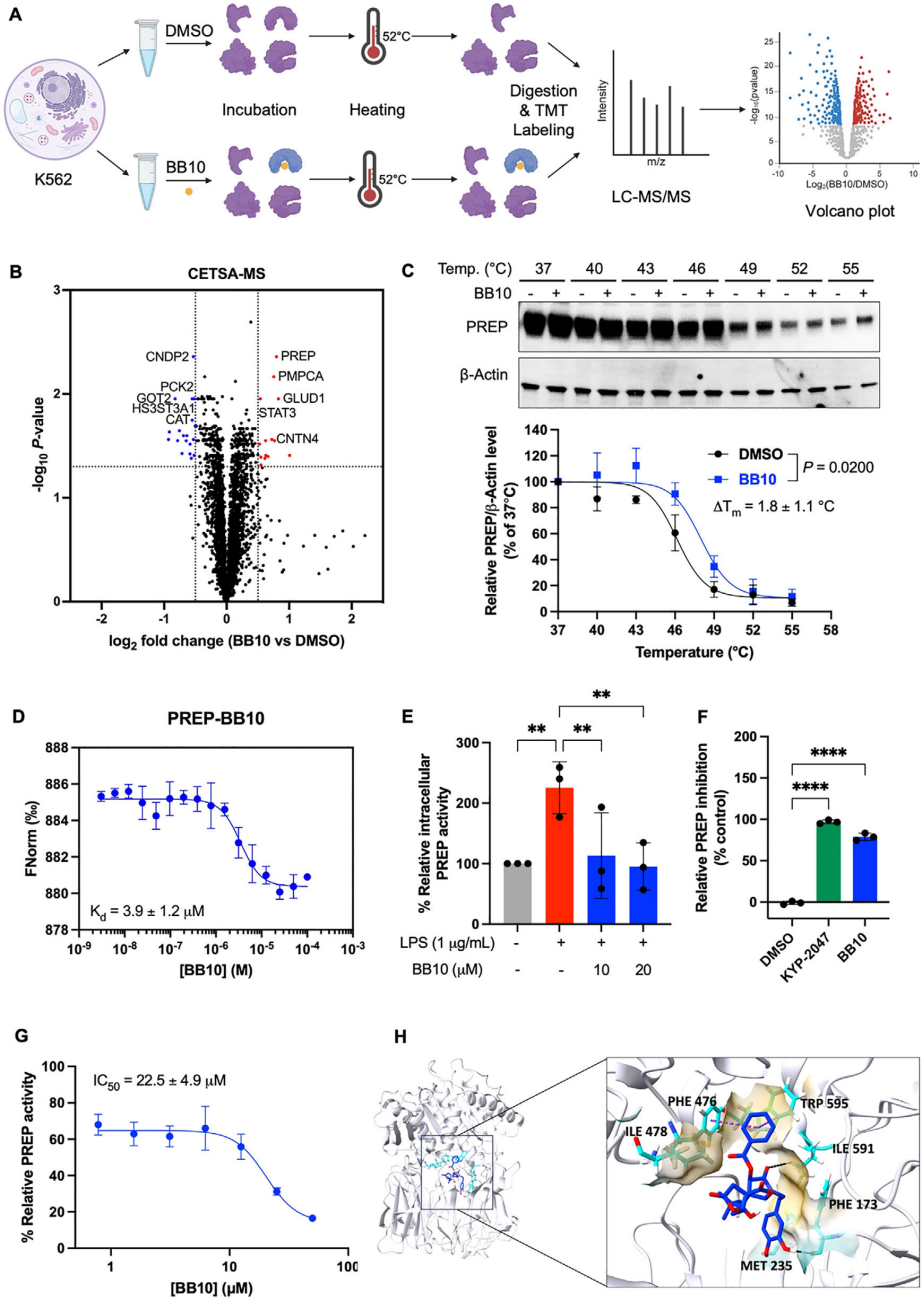
PREP is identified as a molecular target of BB10. (A) Thermal proteome profiling workflow for quantitative chemical proteomic profiling of BB10‐interacting proteins in K562 cell lysates. (B) Volcano plot of CETSA‐MS data, comparing DMSO (0.1%) and **BB10** (20 µm) treatments. Red and blue dots represent thermally stabilized and destabilized proteins, respectively. The top ten proteins with the most significant thermal shifts are labeled. PREP was identified as the top thermally stabilized protein, followed by PMPCA, GLUD1, STAT3, and CNTN4. Thermally destabilized proteins include CNDP2, PCK2, HS3ST3A1, GOT2, and CAT. (C) CETSA‐WB analysis of PREP in LPS‐stimulated BV‐2 microglial cells treated with DMSO (0.1%) or **BB10** (50 µM). Band intensities of PREP were quantified and normalized to β‐actin, and the resulting PREP/β‐actin ratios were used to generate the thermal melting curves shown in the lower panel. **BB10** markedly stabilized PREP against heat‐induced denaturation, resulting in a thermal shift (ΔT_m_) of 1.8 ± 1.1°C. Data are presented as mean ± SD (n = 3). Statistical significance was analyzed using a paired *t*‐test (*p* < 0.05). (D) MST analysis showing the interaction between PREP and **BB10** with K_d_ = 3.9 ± 1.2 µM (n = 3). (E) Intracellular PREP enzymatic activity in LPS‐stimulated BV‐2 cells co‐treated with **BB10** (10 and 20 µM) for 24 h. **BB10** suppressed LPS‐induced PREP hyperactivity. (F) Inhibitory activity of **BB10** (25 µM) against recombinant human PREP enzyme. KYP‐2047 (20 nM) was used as a positive control inhibitor. Data are represented as mean ± SD (n = 3); statistical analysis was performed using one‐way ANOVA followed by Dunnett's post hoc test. ^**^ *p* < 0.01, ^****^ *p* < 0.0001. (G) Dose–response curve showing that **BB10** inhibits PREP enzymatic activity with an IC_50_ of 22.5 ± 4.9 µM (n = 3). (H) Predicted binding mode of **BB10** within the active site of PREP (PDB ID: 9Q62), as determined by molecular docking. Docking analysis was performed using AutoDock Vina. Docking poses were ranked based on predicted binding free energy, and the top‐scoring pose (lowest binding energy) was selected for visualization and analysis in UCSF ChimeraX. The overall structure of PREP is depicted as light grey ribbons (left), with a magnified view of the ligand‐binding pocket shown on the right. **BB10** is represented as blue sticks, while key interacting residues are highlighted as cyan sticks. Hydrogen bonds are indicated by black dashed lines, and π–π stacking interactions by purple dashed lines. The molecular surface of the binding site is colored by hydrophobicity (tan: hydrophobic; light blue: hydrophilic).

We next sought to identify molecular targets that link **BB10** to microglial inflammatory signaling. To elucidate the molecular target of **BB10**, we employed CETSA‐MS (Figure 2A) [45]. CETSA‐MS enables unbiased identification of ligand‐induced thermal stabilization of proteins in cellular systems and has been widely applied for drug target identification [46, 47, 48]. Among multiple candidate proteins stabilized by **BB10** (Table S3), PREP was identified as the most significantly enriched target (*p* = 0.0044; Figure 2B). Follow‐up CETSA combined with Western blotting (CETSA‐WB) confirmed a marked thermal stabilization of PREP, with a change in melting temperature (ΔT_m_) of 1.8°C (*p* = 0.0200; Figure 2C), demonstrating that **BB10** engages PREP in living cells. By contrast, the parental natural product bilobalide did not stabilize PREP as revealed by CETSA‐MS (Figure S1 and Table S4), indicating that **BB10**‐specific structural features are required for PREP engagement. To provide orthogonal biophysical evidence of direct binding, we performed microscale thermophoresis (MST) analysis, which demonstrated that **BB10** binds to PREP with measurable affinity (K_d_ = 3.9 ± 1.2 µm) (Figure 2D). These results establish PREP as a functionally relevant target of **BB10** and support a direct interaction underlying its biological activity.

Neuroinflammatory conditions are known to elevate PREP activity, promoting cytokine release and glial activation [35]. Consistent with this, LPS stimulation increased PREP expression in BV‐2 microglia in a dose‐dependent manner, with significant upregulation at 1 and 2 µg/mL (Figure S5). Importantly, the known PREP inhibitor KYP‐2047 [49] attenuated microglial activation and downregulated *Cox2*, *Il1b*, and *Il6* transcripts (Figures S6), indicating that PREP activity contributes to inflammatory gene expression.

To validate the functional relevance of PREP as a **BB10** target, we assessed how **BB10** influences PREP enzymatic activity under inflammatory conditions. LPS stimulation enhanced PREP enzymatic activity in BV‐2 cells, whereas **BB10** (10 and 20 µm) significantly suppressed this activity (Figure 2E). In a cell‐free recombinant enzyme assay, both **BB10** and the positive control KYP‐2047 inhibited PREP activity (Figure 2F), although **BB10** showed only moderate potency (IC_50_ = 22.5 µm; Figure 2G). These results establish PREP as a molecular target of **BB10** and indicate that PREP inhibition contributes, at least in part, the potent anti‐neuroinflammatory activity of this compound.

Having established PREP as one of the potential targets mediating the anti‐inflammatory action of **BB10**, we next turned to structure‐based optimization to enhance this activity. PREP comprises two distinct domains: a protease catalytic domain with a classical α/β‐hydrolase fold and a seven‐bladed β‐propeller domain [28]. Molecular docking studies predicted that **BB10** preferentially occupies the catalytic pocket of PREP, forming a network of stabilizing interactions. **BB10** is predicted to establish two hydrogen bonds with Phe173 and Ile591, alongside two π–π stacking interactions with Phe476 and Trp595. The hydroxyl groups of **BB10** engage in hydrogen bonding with the carbonyl moiety of Phe173, contributing to stable ligand anchoring within the active site (Figure 2H). These interactions support a model in which **BB10** inhibits PREP by directly engaging the catalytic peptidase pocket.

To experimentally validate this predicted binding mode, we generated a series of site‐directed PREP mutants (PREP^F173A^, PREP^F476A, I478A^, and PREP^I591A, W595A^) targeting residues implicated in ligand interaction. Binding affinities of **BB10** toward these mutants were assessed using MST. Notably, **BB10** exhibited markedly reduced or undetectable binding affinity across all mutant variants (Figure S8) compared to the wild‐type protein (Figure 2D), indicating that these residues are critical for ligand engagement. These findings provide strong experimental support for the docking‐predicted binding interface and reinforce the mechanistic basis of our structure‐activity relationship (SAR) analysis.

### 2.3 Optimizing BB10 Via Rational Chemical Modification Enhances PREP Inhibition

**FIGURE 3:**
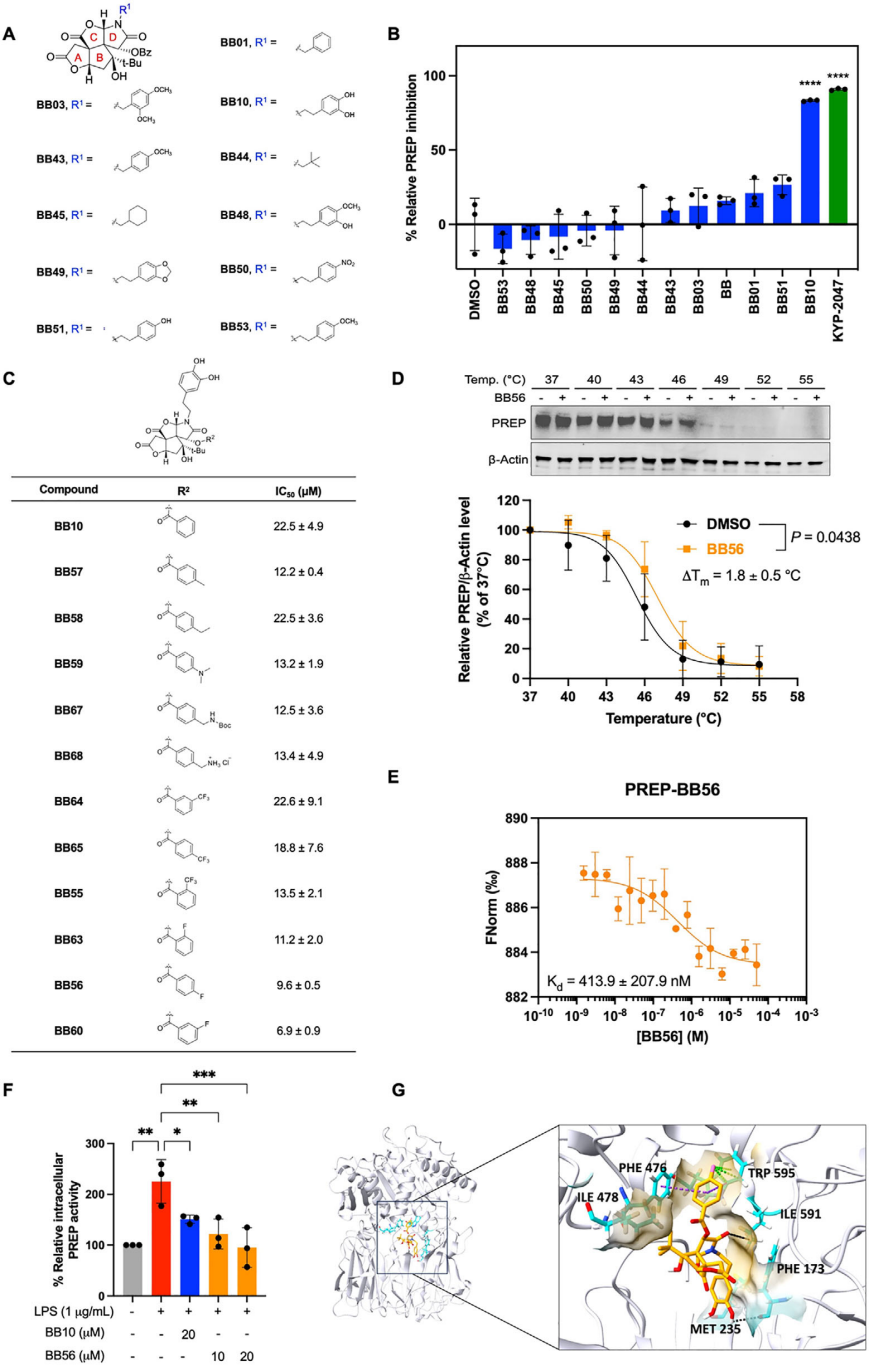
Chemical optimization and structure‐activity relationship for prolyl endopeptidase (PREP) inhibition. (A) Selected chemical structures of screening compounds for PREP inhibition. (B) Screening of PREP inhibitory activity of bilobalide analogues at 25 µM. (C) Structure‐activity relationship of bilobalide analogues and PREP inhibition. (D) CETSA‐WB analysis of PREP in LPS‐stimulated BV‐2 microglial cells treated with DMSO (0.1%) or **BB56** (50 µM). **BB56** markedly stabilized PREP against heat‐induced denaturation, resulting in a thermal shift (ΔT_m_) of 1.8 ± 0.5°C. Data are presented as mean ± SD (n = 3). Statistical significance was analyzed using a paired *t*‐test (*p* < 0.05). (E) MST analysis showing the interaction between PREP and **BB56** with K_d_ = 413.9 ± 207.9 nM (n = 3). (F) Intracellular PREP enzymatic activity in LPS‐stimulated BV‐2 cells co‐treated with **BB10** (20 µM) or **BB56** (10 and 20 µM) for 24 h. **BB56** suppressed LPS‐induced PREP hyperactivity. Data represent mean ± SD (n = 3); statistical analysis was performed using one‐way ANOVA followed by Dunnett's post hoc test. ^*^ *p* < 0.05, ^**^ *p* < 0.01, ^***^ *p* < 0.001, ^****^ *p* < 0.0001. (G) Predicted binding mode of **BB56** within the active site of PREP (PDB ID: 9Q62), as determined by molecular docking. Docking analysis was performed using AutoDock Vina. Docking poses were ranked based on predicted binding free energy, and the top‐scoring pose (lowest binding energy) was selected for visualization and analysis in UCSF ChimeraX. The overall structure of PREP is depicted as light grey ribbons (left), with a magnified view of the ligand‐binding pocket shown on the right. **BB56** is represented as orange sticks, while key interacting residues are highlighted as cyan sticks. Fluorine is represented as pink. Hydrogen bonds are indicated by black dashed lines, π–π stacking interactions by purple dashed lines, and fluorine‐Trp595 interactions by lime dashed lines. The molecular surface of the binding site is colored by hydrophobicity (tan: hydrophobic; light blue: hydrophilic).

Guided by the predicted binding mode of **BB10** to PREP, we pursued systematic structural diversification of R^1^ and R^2^ moieties (Figure 3A–C) to delineate SAR features governing PREP inhibition. Molecular modeling suggested that the D‐ring hydroxyl groups of **BB10** (R^1^ position) engage the key PREP residue (Phe173) through hydrogen‐bonding interactions. To test this hypothesis, we first evaluated the impact of R^1^ modifications on PREP inhibitory activity by varying the position, structure, and number of substituents on the benzyl or phenethyl group, while retaining the benzoyl group at R^2^, as in **BB10** (Figure 3A). Substitution of R^1^ with saturated hydrocarbon groups, such as tert‐butylmethyl (**BB44**) or cyclohexyl (**BB45**), led to a marked loss of PREP inhibition, indicating that aromaticity and/or hydrogen‐bonding capacity at this position are critical for activity. When R^1^ was retained as a phenethyl group (phenethyl‐Ac series), introduction of hydrogen‐bond acceptors at the para position of the benzene ring, such as 3‐hydroxy‐4‐methoxyphenethyl (**BB48**), benzo[d][1,3]dioxol‐5‐yl ethyl (**BB49**), or 4‐nitrophenethyl (**BB50**), nearly abolished PREP inhibitory activity. In contrast, incorporation of a hydrogen‐bond donor at the para position, as in 4‐hydroxyphenethyl (**BB51**), partially retained activity, although with reduced potency relative to the 3,4‐dihydroxyphenethyl substituent in **BB10**. When R^1^ was substituted with a benzyl group (benzyl‐Ac series), analogues bearing hydrogen‐bond acceptors, such as 2,4‐dimethoxybenzyl (**BB03**) and 4‐methoxybenzyl (**BB43**), as well as unsubstituted benzyl (**BB01**), exhibited only modest PREP inhibition. Compared with phenethyl‐Ac series, these benzyl‐Ac series exhibited less pronounced changes in activity but consistently lower inhibitory potency than **BB51** (Figure 3B; and Table S5). Systematic D‐ring diversification established the 3,4‐dihydroxyphenethyl group at R^1^ (**BB10**) as a key determinant for effective PREP engagement and robust enzymatic inhibition.

Based on these findings, subsequent SAR optimization focused on modifying the R^2^ moiety while retaining the 3,4‐dihydroxyphenethyl group at R^1^ (Figure 3C). Introduction of small substituents at the para position of the R^2^ benzene ring, such as methyl (**BB57**) or dimethylamino (**BB59**), resulted in approximately 1.7‐fold improvements in potency, whereas ethyl substitution (**BB58**) had minimal impact. Analogues bearing a (tert‐butoxycarbonyl)amino group (**BB67**) or its deprotected hydrochloride salt (**BB68**) exhibited IC_50_ values of 12.5 and 13.4 µM, respectively. Given the potential metabolic liability of ester linkages, fluorine (**BB56**, **BB60**, **BB63**) and trifluoromethyl (**BB55**, **BB64**, and **BB65**) substituents were introduced at various positions on the R^2^ aromatic ring to explore their impacts on potency. Trifluoromethylated analogues displayed IC_50_ values ranging from 13.5 to 22.6 µM, similar to **BB10** (IC_50_ = 22.5 µM). Notably, fluorinated analogues exhibited enhanced inhibitory potency, with **BB56** and **BB60** emerging as the most active compounds, displaying IC_50_ values of 9.6 and 6.9 µM, respectively. In these analogues, fluorine was introduced at different positions on the aromatic ring of BB10, yielding para‐ (**BB56**), meta‐ (**BB60**), and ortho‐substituted (**BB63**) derivatives. Although **BB60** showed slightly stronger inhibition in the enzymatic assay, the overall PREP inhibitory activities of these fluorinated analogues were comparable (IC_50_ = 6.9–11.2 µM). Considering this narrow activity range, **BB56** was selected as a representative analogue for subsequent biological characterization and mechanistic studies.

To further validate the optimal R^1^ moiety, additional analogues (**BB46**, **BB47**, **BB52**, and **BB54**) were synthesized in which R^2^ is para‐fluorine and R^1^ moiety was altered. Consistent with our SAR hypothesis, these compounds exhibited minimal PREP inhibition (< 25% at 25 µM; Figure S3, lower panel and Table S5), confirming that 3,4‐dihydroxyphenethyl group at R^1^ is critical for PREP inhibition.

Focusing on the optimized analogue **BB56**, cellular target‐engagement and biophysical assays further confirmed its enhanced PREP inhibitory profile. CETSA‐WB analysis demonstrated PREP engagement, as evidenced by significant thermal stabilization (ΔT_m_ = 1.8°C, *p* = 0.0438; Figure 3D). MST measurements revealed that **BB56** binds to PREP with substantially higher affinity (K_d_ = 413.9 ± 207.9 nM) compared to **BB10** (Figures 3E and 2D). Consistently, intracellular PREP activity assays revealed that **BB56** (10–20 µM) suppressed PREP enzymatic activity more effectively than **BB10** in LPS‐stimulated BV‐2 microglia (Figure 3F). Taken together, these results establish the 3,4‐dihydroxyphenethyl group at R^1^ as essential for PREP inhibitory activity, while fluorine substitution at R^2^ further enhances cellular potency, supporting our structure‐activity optimization strategy targeting PREP.

Consistent with these findings, molecular docking analysis provided structural insight into the contribution of fluorine substitution to the enhanced activity of **BB56**. The para‐fluorophenyl group of **BB56** is positioned near the indole ring of Trp595, allowing favorable aromatic/hydrophobic contacts (Figure 3G). The fluorine substituent may fine‐tune local electrostatic and van der Waals complementarity within this aromatic pocket, potentially contributing to the improved binding affinity of **BB56** [50].

### 2.4 BB56 Suppresses Microglial Activation Through Inhibition of PREP

**FIGURE 4:**
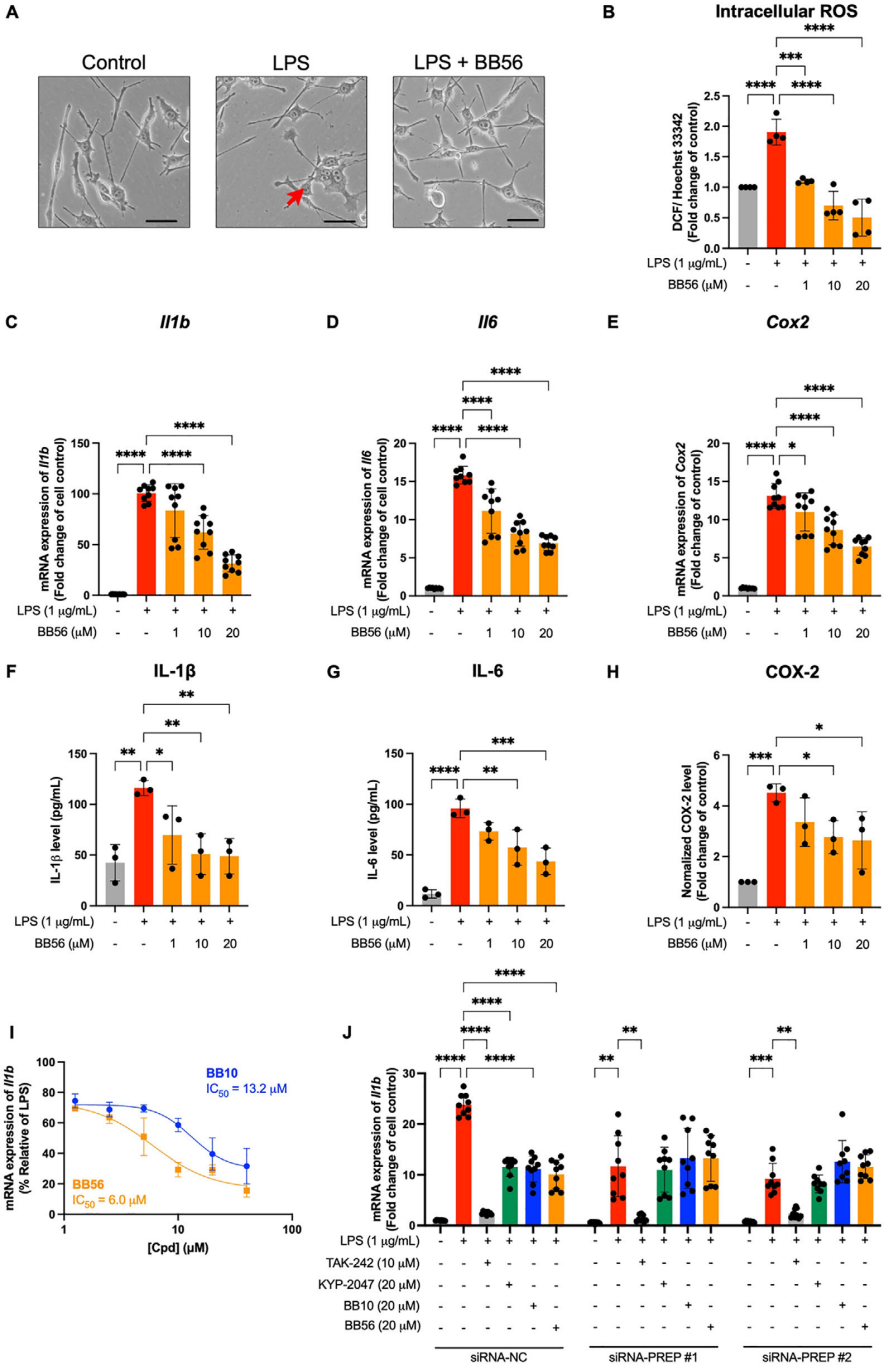
BB56 suppresses microglial activation through inhibition of PREP. (A) Representative microscopic images showing morphological changes in BV‐2 microglia following LPS stimulation (24 h) with or without **BB56** (20 µM). Red arrows indicate activated microglia. 20X magnification, scale bar = 50 µm. (B) Intracellular reactive oxygen species (ROS) levels in LPS‐stimulated BV‐2 cells co‐treated with **BB56** (1, 10, and 20 µM) for 24 h. **BB56** markedly attenuated LPS‐induced ROS production. (C–E) **BB56** suppressed mRNA expression of the pro‐inflammatory genes *Il1b*, *Il6*, and *Cox2* in LPS‐stimulated BV‐2 cells. Cells were co‐treated with LPS and **BB56** for 6 h, followed by RT‐qPCR analysis. (F,G) **BB56** reduced secretion of IL‐1β and IL‐6 cytokines in LPS‐activated BV‐2 cells after 24 h co‐treatment, as determined by ELISA. (H) Western blot analysis showing that **BB56** decreased COX‐2 protein expression in LPS‐stimulated BV‐2 cells after 6 h treatment (refer to original immunoblots in supplementary information Figure S14). (I) Dose‐response curves of **BB10** and **BB56** for inhibition of *Il1b* expression in LPS‐induced BV‐2 cells. (J) *Prep* knockdown abrogated the anti‐inflammatory effects of KYP‐2047, **BB10**, and **BB56**, as indicated by restoration of *Il1b* expression. Data are presented as mean ± SD (n = 3 for panels B, F‐I; and n = 9 for panels C‐E, J). Statistical analysis was performed using one‐way ANOVA (for B‐H) or two‐way ANOVA (for J) followed by Dunnett's post hoc test. ^*^ *p* < 0.05, ^**^ *p* < 0.01, ^***^ *p* < 0.001, ^****^ *p* < 0.0001 (for panels B‐H and J). A paired t‐test was used to analyze the statistical significance of panel I.

To determine whether the enhanced PREP inhibition of **BB56** translates into stronger suppression of microglial activation, we assessed its effects on inflammatory markers in LPS‐stimulated BV‐2 microglia. **BB56** effectively attenuated LPS‐induced morphological transformation of microglia from a ramified to an amoeboid phenotype (Figure 4A) and markedly reduced intracellular ROS production (Figure 4B). Consistently, **BB56** significantly decreased the expression of pro‐inflammatory mediators, including IL‐1β, IL‐6, and COX‐2, at both mRNA (Figure 4C–E) and protein levels (Figure 4F–H). In comparison, **BB56** exhibited stronger anti‐neuroinflammatory activity (IC_50_ = 6.0 µM) than **BB10** (IC_50_ = 13.2 µM), corresponding to a 2.2‐fold enhancement in potency for inhibiting *Il1b* expression in LPS‐induced BV‐2 cells (Figure 4I). These results demonstrate that the enhanced PREP inhibition conferred by **BB56** yields a correspondingly stronger suppression of LPS‐induced microglial inflammatory activation.

To further confirm whether the anti‐neuroinflammatory effects of bilobalide analogues are mediated through PREP inhibition, PREP expression was silenced in BV‐2 cells using specific siRNAs. As shown in Figure S9, PREP‐targeting siRNAs efficiently reduced *Prep* mRNA levels compared to the negative control siRNA (siRNA‐NC). In siRNA‐NC‐transfected cells, LPS markedly upregulated *Il1b*. This upregulation was significantly suppressed by TAK‐242, KYP‐2047, **BB10**, and **BB56**. However, in PREP‐silenced cells, although LPS stimulation still induced *Il1b* expression, only TAK‐242 retained its inhibitory effect. In contrast, KYP‐2047, **BB10**, and **BB56** no longer reduced *Il1b* expression (Figure 4J). These findings demonstrate that PREP plays a pivotal role in LPS‐induced microglial activation and that inhibition of PREP by **BB10** and **BB56** underlies their anti‐neuroinflammatory activity. This evidence supports a mechanistic link between PREP inhibition and the suppression of neuroinflammatory signaling in microglia.

### 2.5 BB56 Suppresses Microglial Inflammatory Signaling Through PREP‐dependent p38 MAPK/ NF‐κB Regulation

**FIGURE 5:**
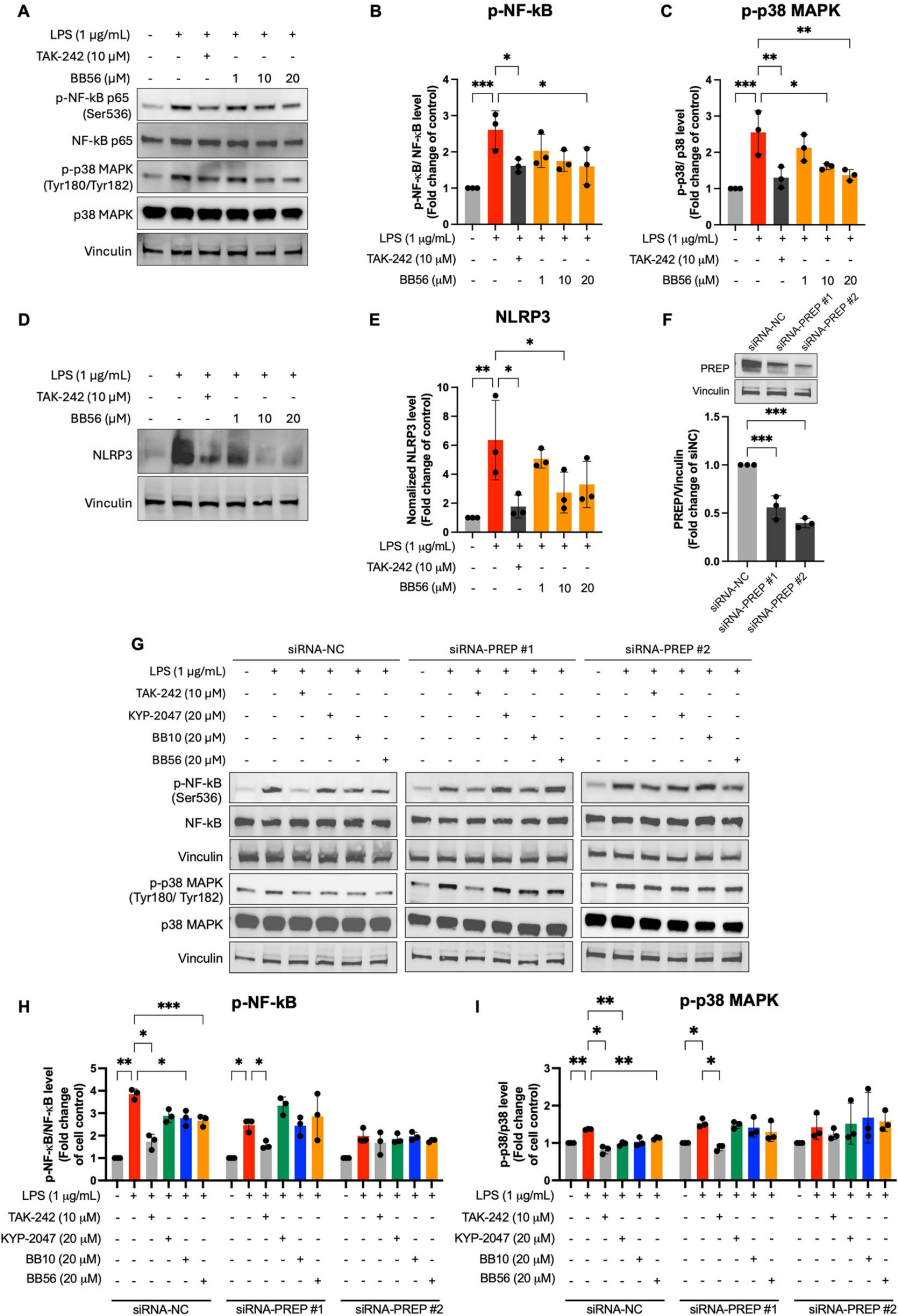
BB56 regulates microglial activation through p38 MAPK/ NF‐κB and NLRP3 inflammasome signaling pathways. (A–C) Representative immunoblots and quantitative analyses of phosphorylated NF‐κB p65 (Ser536), total NF‐κB, phosphorylated p38 MAPK (Thr180/Tyr182), and total p38 MAPK in LPS‐stimulated BV‐2 cells co‐treated with TAK‐242 (10 µM) or **BB56** (1, 10, and 20 µM). **BB56** markedly reduced the phosphorylation of NF‐κB p65 and p38 MAPK after 3 h of treatment. (D,E) Representative immunoblots and quantitative analysis of NLRP3 expression in LPS‐stimulated BV‐2 cells with or without TAK‐242 (10 µM) or **BB56** (1, 10, and 20 µM). **BB56** significantly attenuated LPS‐induced NLRP3 upregulation after 6 h of treatment. (F) Validation of PREP knockdown in BV‐2 microglial cells using siRNA. Two independent siRNA sequences (siRNA‐PREP #1 and #2) effectively reduced PREP protein expression compared with control siRNA. (G–I) Representative immunoblots and quantitative analyses of phosphorylated NF‐κB p65 (Ser536), total NF‐κB, phosphorylated p38 MAPK (Thr180/Tyr182), and total p38 MAPK. PREP knockdown abolishes the inhibitory effects of KYP‐2047, **BB10**, and **BB56** on p38 MAPK activation. In control siRNA‐transfected cells, all compounds suppressed LPS‐induced p38 phosphorylation, whereas in PREP‐knockdown cells, this effect was lost, as evidenced by restored p‐p38 levels, indicating a PREP‐dependent mechanism. Data are presented as mean ± SD (n = 3). Statistical analysis was performed using one‐way ANOVA (for B, C, E, and F) or two‐way ANOVA (for H and I) followed by Dunnett's post hoc test. ^*^ *p* < 0.05, ^**^ *p* < 0.01, ^***^ *p* < 0.001.

To further elucidate the molecular mechanisms underlying the anti‐neuroinflammatory effects of **BB56**, we investigated its impact on key signaling pathways involved in microglial activation, including NF‐κB and p38 MAPK [51, 52]. **BB56** markedly reduced the phosphorylation of NF‐κB (Ser536) and p38 MAPK (Thr180/Tyr182) in LPS‐stimulated BV‐2 cells compared with the vehicle control, indicating suppression of these classical pro‐inflammatory pathways (Figure 5A–C). Given that IL‐1β maturation is tightly regulated by the NLRP3 inflammasome [53], we further examined whether **BB56** influences this pathway. LPS stimulation markedly increased NLRP3 expression, whereas **BB56** significantly reduced this upregulation (Figure 5D,E). These results demonstrate that **BB56** suppresses neuroinflammation by inhibiting the PREP/ p38 MAPK/ NF‐κB signaling cascade and by suppressing IL‐1β production through downregulation of the NLRP3 inflammasome pathway.

To determine whether the suppression of p‐p38 and p‐NF‐κB signaling depends on PREP, we generated PREP‐knockdown BV‐2 microglial cells using siRNA and evaluated the effects of bilobalide‐derived analogues. As shown in Figure 5F, two independent siRNA sequences (siRNA‐PREP #1 and #2) efficiently reduced PREP protein expression compared with control siRNA (siRNA‐NC), confirming effective knockdown. In siRNA‐NC‐transfected cells, treatment with **BB10**, **BB56**, TAK‐242, and KYP‐2047 markedly suppressed LPS‐induced phosphorylation of p38 MAPK and NF‐κB. In contrast, in PREP‐knockdown cells, the inhibitory effects of **BB10**, **BB56**, and KYP‐2047 on p38 and NF‐κB phosphorylation were largely lost, whereas TAK‐242 retained its activity (Figure 5G–I). These findings provide orthogonal biophysical evidence that the suppression of p38 MAPK and NF‐κB signaling by **BB10** and **BB56** is PREP‐dependent, thereby establishing PREP as a functionally relevant molecular mediator of their anti‐inflammatory activity.

### 2.6 BB56 Exhibits a Stronger Neuroprotective Profile than KYP‐2047 in a Zebrafish Model of Neuroinflammation

**FIGURE 6:**
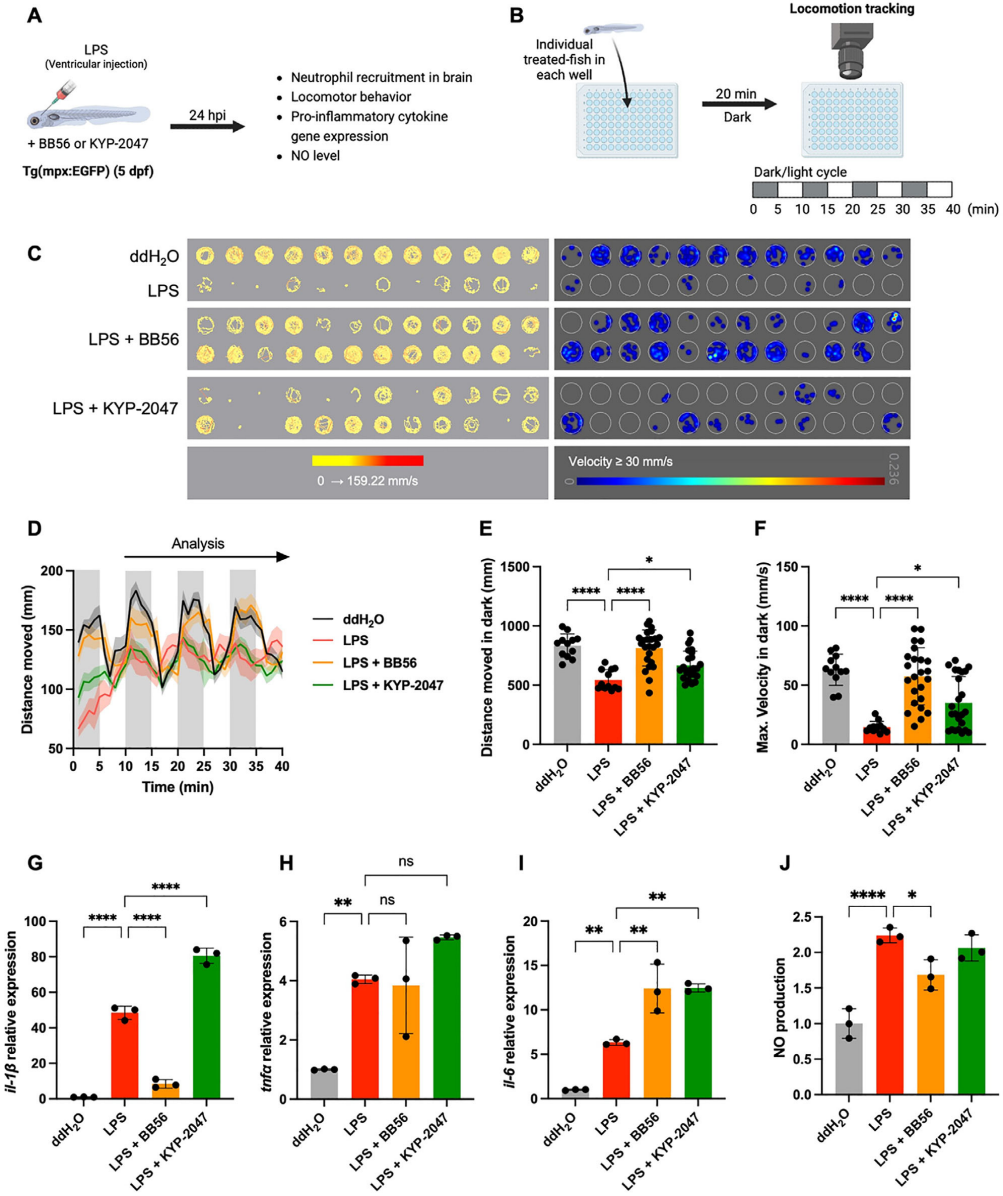
BB56 exhibits neuroprotective activity in a zebrafish model of neuroinflammation. (A) Schematic overview of the zebrafish neuroinflammation model, treatment paradigm, and experimental workflow. Tg(mpx:EGFP) zebrafish larvae at 5 days post‐fertilization (dpf) were intracerebrally injected with LPS (5 mg/mL) or ddH_2_O and immediately treated with **BB56** (50 µM) or the PREP inhibitor KYP‐2047 (100 µM). At 24 h post‐injection (hpi), larvae were randomly selected for fluorescence imaging, locomotor behavior analysis, RT–qPCR, and nitric oxide (NO) quantification. (B) Experimental workflow of locomotion assay. Individual zebrafish larvae were placed into separate wells of a flat‐bottom 96‐well plate. Locomotor activity was recorded using the DanioVision system with EthoVision XT software (Noldus). Prior to recording, larvae were acclimated for 20 min inside the device at 28°C under dark conditions. Locomotion was recorded under alternating dark/light periods (5 min each) to assess stimulus‐evoked responses. Total distance moved was quantified for each larva. (C) Representative trajectories (left) and heatmap (right) showing spatial distribution of high‐speed activity (velocity ≥ 30 mm/s). (D) Distance moved recording of fish under dark (gray shade)/ light (white shade) cycle for 40 min. Locomotion analysis revealed that **BB56** significantly (E) rescued the total distance moved, and (F) increased the maximum swimming velocity during the dark phase in LPS‐stimulated zebrafish. (G) **BB56** significantly suppressed *il‐1β* expression, (H) whereas it did not reduce *tnfα* expression and (I) unexpectedly increased *il‐6* expression in LPS‐challenged larvae. (J) **BB56** also significantly reduced NO production. Data are presented as mean ± SD (n = 12–24 for panels E‐F; and n = 3 for panels G‐J). Statistical analysis was performed using one‐way ANOVA followed by Dunnett's post hoc test. ^*^ *p* < 0.05, ^**^ *p* < 0.01, ^****^ *p* < 0.0001.

To validate the efficacy of **BB56** in a living organism, we assessed its anti‐neuroinflammatory activity in an in vivo zebrafish model of neuroinflammation (Figure 6A,B). Intracerebral LPS injection induced robust neuroinflammation, as evidenced by a marked increase in neutrophil accumulation in the brain (Figure S10). Neither **BB56** nor the known PREP inhibitor KYP‐2047 significantly reduced neutrophil recruitment (Figure S10). It is possible that neutrophil infiltration is not easily reduced by these compounds during our experimental time frame; instead, their protective effect may act downstream of immune cell recruitment.

At the behavioral level, LPS‐challenged larvae exhibited pronounced locomotor deficits (Figure 6C,D), with significantly reduced total distance moved (Figure 6E) and maximum velocity (Figure 6F) relative to ddH_2_O controls. Importantly, while KYP‐2047 partially improved locomotor performance, **BB56** exhibited a more robust functional rescue, significantly enhancing total distance moved (Figure 6E) and maximum velocity (Figure 6F). At the molecular level, LPS markedly increased the expression of *tnfα*, *il‐6*, and *il‐1β*. **BB56** selectively reduced *il‐1β* expression, whereas KYP‐2047 unexpectedly further increased *il‐1β* levels (Figure 6G). No significant changes in *tnfα* expression were detected (Figure 6H), while *il‐6* expression was modestly elevated in zebrafish treated with either **BB56** or KYP‐2047 (Figure 6I). Notably, **BB56**—but not KYP‐2047—significantly reduced LPS‐induced nitric oxide (NO) production (Figure 6J), a hallmark of microglial activation that contributes to neuroinflammation through oxidative stress, mitochondrial dysfunction, and neuronal injury [54].

These in vivo findings are consistent with our observations in LPS‐stimulated BV‐2 microglia, where **BB56** reduced the expression of inflammatory mediators, including NLRP3, a regulator of inflammasome activation and IL‐1β production. Notably, although **BB56** suppressed IL‐6 production in activated BV‐2 microglia, *il‐6* expression was not reduced, and was modestly increased, in the zebrafish model. This discrepancy likely reflects the greater biological complexity of the in vivo system, where multiple cell types, intercellular communication, and regulatory feedback mechanisms contribute to IL‐6 production and homeostasis [55, 56, 57], features largely absent in simplified in vitro cultures.

**FIGURE 7:**
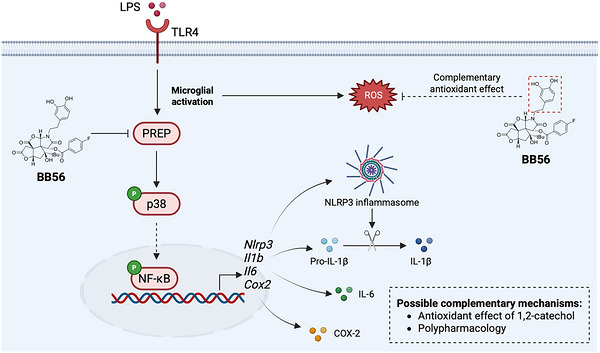
Proposed mechanism of BB56 in suppressing neuroinflammation. Schematic illustration of the PREP‐dependent and complementary mechanisms underlying **BB56**‐mediated anti‐neuroinflammatory activity. **BB56** inhibits microglial activation by targeting PREP, thereby suppressing the downstream p38 MAPK/NF‐κB signaling cascade and reducing the transcription of pro‐inflammatory mediators (*Il1b*, *Il6*, and *Cox2*). In parallel, **BB56** attenuates NLRP3 inflammasome activation, limiting the maturation of pro‐IL‐1β to IL‐1β. Additionally, the 1,2‐catechol moiety of **BB56** may contribute a complementary antioxidant effect by reducing intracellular ROS levels, suggesting a multifaceted mode of action.

Overall, the concordant suppression of inflammatory mediators across in vitro and in vivo models, together with improved locomotor performance, indicates that **BB56** confers functional neuroprotection through modulation of inflammatory signaling outputs. Importantly, although **BB56** is less potent than KYP‐2047 in enzymatic PREP inhibition assays, it exhibited superior in vivo anti‐neuroinflammatory efficacy. This observation suggests that mechanisms beyond PREP inhibition may contribute to its overall activity. In particular, the 1,2‐catechol moiety present in **BB56** may confer antioxidant properties, which could partially influence the observed anti‐inflammatory effects. Together, these findings suggest that the biological activity of **BB56** may arise from the combined contribution of PREP inhibition and additional complementary mechanisms (Figure 7).

## 3 Conclusions

In summary, molecular editing of a natural product scaffold enabled target discovery and rational optimization of a PREP‐inhibitory chemotype that suppresses microglial inflammatory signaling. CETSA‐MS, together with orthogonal validation, identified PREP as a cellular target engaged by bilobalide‐derived lactam analogues. The lead compound **BB10** and its SAR‐optimized fluorine derivative **BB56** inhibit PREP at micromolar concentrations and consequently attenuate the p38 MAPK/ NF‐κB signaling cascade and NLRP3 inflammasome activation in LPS‐stimulated microglia (Figure 7). Consistent with these mechanistic findings, **BB56** significantly reduced neuroinflammation‐associated readouts and improved functional outcomes in an in vivo zebrafish model. Given the established involvement of PREP in neurodegenerative disorders, including Alzheimer's disease [58, 59] and ischemic stroke [60, 61], our results highlight PREP‐targeting chemotypes as promising candidates for disease‐modifying intervention. The ability of **BB56** to broadly suppress microglial activation and downstream inflammatory signaling further supports its potential as a neuroprotective agent, with possible utility as an adjunct to existing therapies and broader applicability across neurodegenerative conditions.

Notably, although **BB56** is ∼100‐fold less potent than the widely used PREP inhibitor KYP‐2047 in enzymatic assays, it unexpectedly exhibited stronger functional rescue in vivo. Combined with our CETSA‐MS data, this suggests that **BB56** may act through additional targets beyond PREP, pointing to a potential polypharmacological mechanism. Nevertheless, these results establish a molecular editing‐enabled, natural product chemotype for PREP inhibition and provide a mechanism‐defined starting point for developing microglia‐modulating compounds with in vivo efficacy.

Several repurposed agents, including desloratadine, DW140006, fluoxetine, and minocycline, have been reported to modulate microglial activation, and more selective inhibitors such as the NLRP3 inhibitor inzomelid are under development for inflammation‐related disorders, including Parkinson's disease [62, 63, 64, 65, 66]. However, many of these approaches primarily target a single pathway, which may limit their ability to address the networked and multifactorial nature of neuroinflammation. In contrast, our bilobalide lactam analogues (e.g., **BB10**) suppress multiple convergent neuroinflammatory nodes downstream of PREP and also mitigate ferroptotic cell death in neuronal and neuroimmune cells [11]. These findings support the feasibility of using rational molecular editing to access chemotypes with broader microglia‐modulating and potentially neuroprotective profiles.

Despite these encouraging findings, several limitations should be acknowledged. First, the anti‐neuroinflammatory effects of **BB56**‐derived PREP inhibitors were assessed primarily in the BV‐2 microglial cell line and a zebrafish neuroinflammation model. In the future, validation in primary microglia, human iPSC‐derived microglia, and in vivo mammalian models will further strengthen these initial findings. Second, although CETSA‐MS and siRNA‐based experiments implicate PREP as a functionally relevant molecular target, comprehensive selectivity profiling across related serine proteases, together with high‐resolution structural studies, will help to precisely define the binding mode and to minimize potential off‐target liabilities. Finally, while fluorination improved PREP inhibition, the pharmacokinetic properties, brain penetration, and safety profile of **BB56** remain to be fully characterized. Conducting integrated in vivo efficacy, pharmacokinetic, and SAR investigations will help further advance **BB56** and related analogues toward translational neuroprotective drug development.

## 4 Experimental Section

### 4.1 Cell Culture

BV‐2 mouse microglial cells were obtained from The World Cell Factory (Cat. No. iCell‐m011, iCell Bioscience Inc, Shanghai, China; RRID: CVCL_0182) and maintained in Dulbecco's Modified Eagle Medium (DMEM) supplemented with 10% fetal bovine serum and 1% penicillin–streptomycin. Cells were cultured at 37°C in a humidified incubator with 5% CO_2_. Routine subculturing was performed every 2–3 days using 0.25% trypsin–EDTA to ensure optimal growth and viability. Cell line used in this study was contamination‐free.

### 4.2 Intracellular ROS Assay

BV‐2 cells (5 × 10^4^ cells/well) were cultured in a 96‐well black plate with a clear bottom and grown overnight. The cells were co‐treated with LPS (1 µg/mL) and bilobalide analogues (1, 10, and 20 µM) for 24 h. The treatment was removed and replaced with CM‐H_2_DCFDA (1 µM; Invitrogen, OR USA) and Hoechst 33342 (10 µg/mL) for 45 min, then the cells were washed twice with DPBS. The fluorescence intensity of intracellular ROS (excitation/emission–485/535 nm) and nuclear staining (excitation/emission–360/465 nm) was measured using a microplate reader. The intensity of intracellular ROS staining was normalized to nuclear staining.

### 4.3 RNA Extraction and RT‐qPCR Analysis

BV‐2 cells were seeded into a 6‐well plate at a cell density of 2 × 10^5^ cells per well and incubated overnight. Cells were treated with media (cell control), 1 µg/mL LPS + 0.1% DMSO (inducer control), or 1 µg/mL LPS + bilobalide analogue (treatment). After 6 h of treatments, the cells were washed twice with PBS, and total RNA was extracted using RNA Fast 200 RNA extraction kit (Fastagen, China), followed the manufacturing's instruction. The total RNA concentration was determined using NanoDrop 2000 spectrophotometer (Thermo Scientific, MA, US). The RNA was complementary reverse‐transcribed into cDNA using PrimeScript RT Master Mix (Takara Bio, Japan). The cDNA was used as a template for qPCR amplification using TB Green Premix Ex Taq (Til RNase H Plus) (Takara Bio, Japan) on the Applied Biosystems QuantStudio 12K Flex Real‐Time PCR System (Thermo Fisher Scientific, MA, US) with specific primer pairs (refer to Table S1). The relative gene expression was analyzed using the ΔΔ C_t_ method.

### 4.4 Enzyme‐linked Immunosorbent Assay (ELISA)

BV‐2 cells were seeded at a cell density of 3 Í10^5^ cells/well in a 6‐well plate for overnight and then treated with the LPS (1 µg/mL) in the presence or absence of bilobalide analogue for 24 h. The cell culture media were collected and centrifuged at 10 000×g for 10 min to eliminate cell debris. The cell culture supernatants were used to measure the levels of IL‐1β, and IL‐6 using mouse IL‐1β, and IL‐6 uncoated ELISA kits (Invitrogen, Thermo Fisher Scientific), respectively. These assays were performed according to the manufacturer's instructions.

### 4.5 Protein Extraction and Western Blotting

BV‐2 microglial cells were seeded at a density of 2 × 10^5^ cells per well in 6‐well plates and allowed to adhere overnight. Cells were then treated under the same conditions described above for the indicated time points. Following treatment, cells were washed twice with PBS and lysed in Pierce RIPA buffer (Thermo Scientific) supplemented with a protease inhibitor cocktail (Roche). Lysates were centrifuged at 13 000 rpm for 20 min at 4°C, and the supernatants were collected as total protein extracts. Protein concentrations were quantified using the Pierce BCA Protein Assay Kit (Thermo Scientific). Equal amounts of protein were separated on FuturePAGE precast gels (ACE Biotechnology, China) using MOPS‐SDS running buffer (ACE Biotechnology). PageRuler Prestained Protein Ladder (Thermo Fisher Scientific) served as the molecular weight marker. Proteins were transferred onto 0.2‐µm PVDF membranes (Thermo Scientific) and blocked with blocking buffer (ACE Biotechnology). Membranes were incubated with specific primary antibodies (refer to Table S2) overnight at 4°C, followed by HRP or DyLight 488‐conjugated secondary antibodies (refer to Table S2) for 1 h at room temperature. Protein bands were visualized with the Ultra High Sensitivity ECL Kit (MedChemExpress, NJ, USA) for HRP‐secondary antibodies and imaged using the ChemiDoc MP Imaging System (Bio‐Rad, CA, USA). Band intensities were quantified using ImageJ software (NIH, MD, USA).

### 4.6 CETSA

#### 4.6.1 CETSA‐MS

CETSA‐MS was initially performed in the chronic myeloid leukemia K562 cell line. K562 cells were selected because they are well‐established for proteome‐wide CETSA studies, offering robust protein expression levels and high reproducibility for thermal proteome profiling. This platform allows efficient identification of ligand‐induced thermal stabilization across the cellular proteome [67, 68]. Our CETSA‐MS workflow was performed according to a previous study (Figure 2A) [67]. Briefly, K562 cells were lysed by three freeze‐thaw cycles in liquid nitrogen. The resulting cell lysates were pelleted by centrifugation at 20 000 × g for 20 min at 4°C, and the supernatant was collected. K562 cell lysates are treated with 20 µM BB10 (final concentration) and DMSO as vehicle control, followed by incubation at RT for 10 min. After heating at 52°C, the soluble protein fraction was isolated by centrifugation at 20 000 x g for 20 min at 4°C. Proteins from the soluble fractions of drug‐treated and control samples were reduced, alkylated, and digested with trypsin. The resulting peptides were labeled using Tandem Mass Tag (TMT) 10‐plex reagents according to the manufacturer's instructions, with three biological replicates for each condition.

The TMT‐labeled samples were pooled and fractionated by C18 StageTip‐based high‐pH reversed‐phase fractionation according to a previous study [67]. The fractions were analyzed by LC‐MS/MS on an Orbitrap Exploris 480 mass spectrometer. The raw data was processed and searched with Thermo Scientific Proteome Discoverer (version 3.1) using the SEQUEST HT search engine. The output results from Proteome Discoverer were used to generate volcano plots using Python (version 3.9). Proteins exhibiting a significant stabilization in the BB10‐treated samples at 52°C were classified as putative targets.

#### 4.6.2 CETSA‐WB

To generate the disease‐related model, BV‐2 cells were seeded in 10 cm cell culture dishes and allowed to adhere overnight, then the cells were exposed to LPS for 24 h. After that, the cells were treated with 0.1% DMSO or 50 µM of bilobalide analogues for 1 h. After incubation, cells were collected by trypsinization and washed 3 times with PBS containing a protease inhibitor at room temperature. The cell numbers were then counted and adjusted. Cells were then aliquoted and heated at different temperatures for 3 min and then immediately frozen in liquid nitrogen. Repeated freeze/ thaw cycles 3 times were performed to get lysate. The cell debris and aggregates were then removed by centrifugation. The supernatant was mixed with loading buffer and heated at 95°C for 5 min. The protein level was then measured using Western blotting assay. Band intensity was normalized using ImageJ software (NIH, MD, USA). Calculation of ΔT_m_ (change in melting temperature) is: ΔT_m_ = T_m_ (ligand‐bound)—T_m_ (unbound).

### 4.7 PREP Enzymatic Assay

Human PREP‐His tag recombinant protein (20 ng) was mixed with the tested compound in a 96‐well black plate and incubated at RT for 10 min. Then, the fluorogenic substrate Suc‐Gly‐Pro‐AMC (MedChemExpress) was added to each well at a final concentration of 25 µM. The assay buffer contained 10 mM Tris‐HCl (pH 7.4), 10 mM MgCl_2_ and 0.05% Tween 20. The fluorescent intensity was measured at an excitation/emission wavelength of 380/460 nm every 5 min for 1 h. Percent relative activity was calculated from the ratio of fluorescent intensity produced by time, compared to the enzyme control (no inhibitor).

### 4.8 Expression and Purification of Recombinant PREP and Mutant Proteins

The pGEX6p1‐PREP plasmid (Addgene plasmid #179387; http://n2t.net/addgene:179387; RRID:Addgene_179387) [69] was transformed into *Escherichia coli* BL21 competent cells for recombinant protein expression. PREP mutant constructs (PREP^F173A^, PREP^F476A, I478A^, and PREP^I591A, W595A^) were synthesized (TsingKe, China) based on the wild‐type pGEX6p1‐PREP backbone. Protein expression was induced with 0.2 mM isopropyl β‐D‐1‐thiogalactopyranoside (IPTG) at 20°C for 16 h. Cells were harvested by centrifugation at 8000 × g for 10 min at 4°C and resuspended in pre‐chilled lysis buffer (50 mM Tris‐HCl, pH 8.0, 500 mM NaCl, 2 mM dithiothreitol (DTT), 0.2 mM benzamidine, and 10% glycerol). The cell suspension was maintained on ice and lysed by sonication. Cell debris was removed by centrifugation at 40 000 × g for 1 h at 4°C, and the clarified supernatant was filtered prior to purification. The lysate was then purified using glutathione‐S‐transferase affinity chromatography under gentle rotation for 2 h at 4°C. The immobilized beads were extensively washed with lysis buffer to remove non‐specifically bound proteins. The GST tag was removed by adding approximately 100 µg of PreScission protease, followed by incubation at 4°C for 16 h. The cleaved PREP protein was subsequently eluted using elution buffer (50 mM Tris‐HCl, pH 8.0, 200 mM NaCl, and 10% glycerol). To further improve purity, the eluted protein was subjected to size‐exclusion chromatography. Fractions corresponding to monomeric PREP were collected, analyzed by SDS‐polyacrylamide gel electrophoresis (SDS‐PAGE), and concentrated for subsequent experiments.

### 4.9 Microscale Thermophoresis (MST) Analysis

Purified PREP protein was fluorescently labeled using the Monolith NT Protein Labeling Kit RED‐NHS (NanoTemper Technologies) according to the manufacturer's instructions. Labeled PREP was diluted to a final concentration of 20 nM and incubated with a 16‐point serial dilution of **BB10** or **BB56** (final concentration range: 3 nM to 200 µM) in MST assay buffer at a 1:1 volume ratio. MST measurements were performed using a Monolith NT.115 instrument (NanoTemper Technologies) at 22°C, using 70% excitation power and 40% MST power. Binding affinities (dissociation constants, K_d_) were determined by fitting the thermophoresis data using MO. Affinity Analysis software (NanoTemper Technologies).

### 4.10 PREP Activity Assay in BV‐2 Microglial Cells

BV2 microglial cells were seeded into black 96‐well plates with clear bottoms at a density of 1 × 10^4^ cells per well and incubated overnight. The cells were then stimulated with LPS (1 µg/mL) in the presence or absence of the bilobalide analogue at 37°C for 1 h. Following stimulation, the Suc‐Gly‐Pro‐AMC fluorogenic substrate was added to each well at a final concentration of 50 µM to initiate the enzymatic reaction. Fluorescence, representing PREP catalytic activity, was measured every 5 min for 1 h at an excitation/emission wavelength of 380/460 nm. PREP activity was quantified by calculating the slope of the fluorescence intensity vs. time curve, expressed as a percentage of the untreated cell control.

### 4.11 Silencing of PREP in BV‐2 Cells

BV cells (2 × 10^5^ cells) were seeded in a 6‐well plate overnight. The cells were transfected with siRNA‐PREP #1 (5’‐CCGCCGUACAGGAUUAUCATT‐3’/5’‐UGAUAAUCCUGUACGGCGGTT‐3’), siRNA‐PREP #2 (5’‐GGGUGACAAUCAAGUUCAUTT‐3’/5’‐AUGAACUUGAUUGUCACCCTT‐3’), or siRNA‐negative control (siRNA‐NC) for 24 h using Lipomaster 2000 Transfection Reagent (Vazyme, China). After transfection, the cells were treated with LPS (1 µg/mL) and bilobalide analogues (20 µM) for 6 h. Total RNA was extracted, and RT‐qPCR analysis was performed as described above.

### 4.12 Molecular Docking

The crystal structure of PREP (Protein Data Bank ID: 9Q62) [70] was retrieved from the Protein Data Bank and prepared using Open Babel [71]. All water molecules and heteroatoms were removed, and polar hydrogen atoms were added to the protein structure prior to docking. The three‐dimensional structures of **BB10** and **BB56** were generated and converted into SDF format for docking simulations. To identify potential ligand‐binding sites without prior bias, initial blind docking was performed using DiffDock [72]. The prepared PREP structure and ligand molecules (**BB10** and **BB56**) were used as inputs for the simulations.

Binding poses were ranked based on DiffDock confidence scores, and the top‐scoring conformations (−1.42 for PREP‐**BB10** and −1.29 for PREP‐**BB56**) were selected to define the primary binding regions. These initial docking results were subsequently refined using AutoDock Vina [73] to improve atomic‐level interaction modeling and estimate binding affinities. Final binding poses were selected based on the lowest predicted binding free energies (−9.2 kcal mol^−^^1^ for PREP‐**BB10** and −8.5 kcal mol^−^^1^ for PREP‐**BB56**). The resulting PREP‐ligand complexes were visualized using UCSF ChimeraX [74]. Protein‐ligand interactions, including hydrogen bonding, hydrophobic contacts, and π–π stacking interactions, were analyzed to validate the proposed binding modes.

### 4.13 Investigation of Anti‐Neuroinflammation in Zebrafish Model

#### 4.13.1 Zebrafish Maintenance

Adult zebrafish were maintained in a recirculating aquatic system at 28°C under a 14 h light / 10 h dark photoperiod and fed twice daily. Embryos were obtained via standard breeding protocols [75]. Larvae were raised in embryo medium consisting of 0.03% Instant Ocean and 0.0002% methylene blue in reverse osmosis‐distilled water. At 1 day post‐fertilization (dpf), the culture medium was supplemented with 0.0045% 1‐phenyl 2‐thiourea (PTU, Sigma–Aldrich) to inhibit melanin formation. All experimental procedures were conducted in accordance with the Guide for the Care and Use of Laboratory Animals [76] and were approved by the Department of Science and Technology of Zhejiang Province, China. Experiments were performed at Cipher Gene, Ltd, under the institutional laboratory animal use license (SYXK (Zhe) 2024‐0021).

#### 4.13.2 Construction of the Zebrafish Neuroinflammation Model

Tg(mpx:EGFP) and wild‐type TU zebrafish were utilized to construct the neuroinflammation model as previously described [77]. Briefly, 5 dpf zebrafish larvae were anesthetized with 150 µM pancuronium (Sigma–Aldrich), and ventricular injection was performed using an injection volume of 1 nL. The experimental group received 5 mg/mL LPS (MedChemExpress), whereas the control group received ddH_2_O. Compounds were applied to the culture medium immediately after the injection, using **BB56** (50 µM) or KYP‐2047 (100 µM). Compound working concentrations were determined in a 48‐h exposure toxicity assay, and the maximum non‐toxic concentrations were used in subsequent experiments. At 24 h post‐injection (hpi), larvae were randomly picked for imaging, locomotion behavioral assay, RT‐qPCR, and NO quantification (Figure 6A).

#### 4.13.3 Locomotion Behavioral Study

Individual zebrafish larvae were placed into separate wells of a flat‐bottom 96‐well plate containing 200 µL of embryo medium. Locomotor activity was recorded using the DanioVision system with EthoVision XT software (Noldus). Prior to recording, larvae were acclimated for 20 min inside the device at 28°C under dark conditions. Locomotion was recorded under alternating dark/ light periods (5 min each) to assess stimulus‐evoked responses. Total distance moved was quantified for each larva. As the response had not fully stabilized during the initial cycle, data from the first dark/ light cycle were excluded from the analysis (Figure 6B).

#### 4.13.4 RNA Extraction and RT‐qPCR in Zebrafish Model

Zebrafish embryos were selected from each experimental group, with ten larvae per group and three biological replicates. Total RNA was extracted using the RNA‐easy Isolation Reagent (Vazyme Biotech Co., Ltd., Nanjing, China) following the manufacturer's protocol. cDNA synthesis was performed using the HiScript III first Strand cDNA Synthesis Kit (+gDNA wiper) (Vazyme Biotech Co., Ltd.). RT‐qPCR was carried out using Applied Biosystems QuantStudio 1 (ThermoFisher) and AceQ qPCR SYBR Green Master Mix (Vazyme Biotech Co., Ltd.). Primer sequences used were as follows: for *tnfα* forward 5′‐ GCTTATGAGCCATGCAGTGA ‐3′, reverse 5′‐ TGCCCAGTCTGTCTCCTTCT ‐3′; for *il‐1β* forward 5′‐ TGTGTGTTTGGGAATCTCCA‐3′, reverse 5′‐ CTGATAAACCAACCGGGACA ‐3′; for *il‐6* forward 5′‐ TCAACTTCTCCAGCGTGATG ‐3′, reverse 5′‐ TCTTTCCCTCTTTTCCTCCTG ‐3′. Gene expression levels were quantified using the ΔΔCt method, normalizing target gene expression to the housekeeping gene β‐actin.

#### 4.13.5 NO Assay

For NO measurement, 20–30 larvae per group were collected, with three biological replicates. Larvae were placed in 1.5 mL centrifuge tubes with 200 µL PBS, homogenized, and centrifuged at 10 000 rpm for 5 min. Supernatants were collected, and NO levels were measured using the Griess method with a commercial Nitric Oxide Assay Kit (Beyotime, Shanghai, China).

### 4.14 Statistical Analysis

Data are presented as mean ± standard deviation (SD) from at least three independent experiments. The sample size (n) for each analysis is indicated in the corresponding figure legends. Statistical analyses were performed using one‐way ANOVA followed by Dunnett's post hoc test for multiple comparisons, or a paired Student's *t*‐test for comparisons between two groups, as appropriate. All tests were two‐sided, and a *p* < 0.05 was considered statistically significant. Statistical analyses and graph generation were performed using GraphPad Prism (version 10.2.1).

## Author Contributions

C.S. and B.W.L.N. designed and conceived the study. C.S., Y.Q., and X.H. performed biological experiments. W.W. and S.S. performed chemical experiments. Q.W., Y.L., and J.S. performed the CETSA‐MS experiment. X.Y. and J.L. performed zebrafish experiments. L.Y., K.S., C.W.Y., and S.W.N.A. performed structural biology experiments. C.S., Y.Q., W.W., and B.W.L.N. drafted the manuscript. C.S., Y.Q., W.W., S.S., J.L., J.H., Z.C., X.L., Y.G., S.W.N.A., B.I., H.K., and B.W.L.N. revised the manuscript. B.W.L.N. supervised the research, acquired funding, and is the primary contact for requests for materials. All authors have given approval to the final version of the manuscript.

## Conflicts of Interest

The authors declare the following competing financial interest(s): B.W.L.N., C.S., Y.Q., W.W., and S.S. are inventors on patent applications submitted by The Chinese University of Hong Kong covering the analogues described in this manuscript. B.W.L.N. is a Scientific Founder and shareholder of Antropy Therapeutics Limited.

## Supporting information




**Supporting File**: advs76744‐sup‐0001‐SuppMat.docx.

## Data Availability

All data supporting the findings of this study are available within the article and its supplementary materials. Requests for further information can be addressed to and will be provided by the corresponding authors.
