# Supplementary material for: Molecular Editing Reveals a Bilobalide Chemotype that Attenuates Prolyl Endopeptidase (PREP)‐Linked Neuroinflammation
Source: Adv Sci (Weinh). 2026 Aug 11:e00011. Online ahead of print. doi: 10.1002/advs.202600011 (PMC13460414; doi:10.1002/advs.202600011)
Supplement: Supplementary file 1 [file ADVS-9999-e00011-s001.docx]

*Supporting Information for*

**Molecular Editing Reveals a Bilobalide Chemotype that Attenuates Prolyl Endopeptidase (PREP)-Linked Neuroinflammation**

Chanin Sillapachaiyaporn^1, 2, ⊥^, Wenjing Wang^1, 2, ⊥^, Yao Qin^1, 2, ⊥^, Lili Yan^1,2^, Stephan Scheeff^1^, Xu He^1^, Qiong Wu^3^, Yue Lin^3^, Jinsai Shang^3^, Xin Ye^4^, Jing Liu^4^, Kailei Sun^5^, Chun Wai Yeung^5^, Junzhe Huang^2, 8^, Zhenghui Chen^9^, Xiaodong Liu^2, 6, 7^, Yiqun Geng^9^, Shannon Wing Ngor Au^5^, Bonaventure Y. Ip^2, 8, 10^, Ho Ko^2, 8, 10^, Billy Wai-Lung Ng^1, 2, 7, 10, *^

^1^Guangdong-Hong Kong-Macao Joint Laboratory for New Drug Screening, School of Pharmacy, Faculty of Medicine, The Chinese University of Hong Kong, Shatin, Hong Kong SAR, China

^2^Li Ka Shing Institute of Health Sciences, Faculty of Medicine, The Chinese University of Hong Kong, Shatin, Hong Kong SAR, China

^3^School of Basic Medical Sciences, Guangzhou National Laboratory, State Key Laboratory of Respiratory Disease, Guangzhou Medical University, Guangzhou 511436, China

^4^Cipher Gene Ltd, Chengdu, China

^5^Center for Protein Science and Crystallography, School of Life Sciences, The Chinese University of Hong Kong, Shatin, Hong Kong, China

^6^Department of Anaesthesia and Intensive Care, Faculty of Medicine, The Chinese University of Hong Kong, Shatin, Hong Kong SAR, China

^7^Peter Hung Pain Research Institute, Faculty of Medicine, The Chinese University of Hong Kong, Shatin, New Territories, Hong Kong SAR, China

^8^Department of Medicine and Therapeutics, Faculty of Medicine, The Chinese University of Hong Kong, Shatin, Hong Kong SAR, China

^9^State Key Laboratory of Bioactive Substance and Function of Natural Medicines, Beijing Key Laboratory of Active Substances Discovery and Druggability Evaluation, Institute of Materia Medica, Chinese Academy of Medical Sciences and Peking Union Medical College, Beijing 100050, China

^10^Gerald Choa Neuroscience Institute, The Chinese University of Hong Kong, Shatin, Hong Kong SAR, China

^⊥^Authors contributed equally

*Corresponding author, e-mail: billyng@cuhk.edu.hk

Table of Contents

[1. Biology Experiments	1](#_Toc228559216)

[1.1. General Methods	1](#_Toc228559217)

[1.1.1. Treatment preparation	1](#_Toc228559218)

[1.1.2. Cytotoxicity	1](#_Toc228559219)

[1.1.3. Cell morphology evaluation	1](#_Toc228559220)

[1.1.4. Quantitative real-time PCR (qPCR) analysis	1](#_Toc228559221)

[1.1.5. Western blotting	2](#_Toc228559222)

[1.1.6. Fluorescence imaging of neutrophil recruitment in zebrafish	3](#_Toc228559223)

[1.2. Supplementary Tables/ Figures	3](#_Toc228559224)

[1.2.1. Cellular thermal shift assay–mass spectrometry (CETSA-MS) data	3](#_Toc228559225)

[1.2.2. Cellular thermal shift assay–Western blot (CETSA-WB) of **BB10** and identified targets from CETSA-MS	6](#_Toc228559226)

[1.2.3. Chemical structures of D-ring modification	8](#_Toc228559227)

[1.2.4. Screening of PREP inhibitory activity of bilobalide analogues	8](#_Toc228559228)

[1.2.5. Cytotoxicity of **BB10** and **BB56** against BV-2 microglia cells	9](#_Toc228559229)

[1.2.6. LPS activates prolyl endopeptidase (PREP) protein expression	9](#_Toc228559230)

[1.2.7. Inhibition of PREP by KYP-2047 suppresses BV-2 microglial inflammation	10](#_Toc228559231)

[1.2.8. Dose-response curves of bilobalide analogues for PREP inhibitory activity	11](#_Toc228559232)

[1.2.9. Quantitative microscale thermophoresis (MST) analysis of **BB10** and **BB56** binding to PREP mutants	13](#_Toc228559233)

[1.2.10. Prep silencing in BV-2 cells by siRNA	14](#_Toc228559234)

[1.2.11. Effects of **BB56** on neutrophil recruitment in a zebrafish model of neuroinflammation	14](#_Toc228559235)

[1.2.12. Original Western blots	15](#_Toc228559236)

[2. Chemical Experiments	28](#_Toc228559237)

[2.1. Chemical Synthesis	28](#_Toc228559238)

[2.1.1. General Chemical Experimental Information	28](#_Toc228559239)

[2.1.2. General method A: synthesis of diBz-Bilobalide analogues **BB37** to **BB42**, **M-10** to **M-12**	28](#_Toc228559240)

[2.1.3. General method B: synthesis of D-ring analogues **BB43** to **BB54**	34](#_Toc228559241)

[2.1.4. General method C: synthesis of **BB55** to **BB60**, **BB63** to **BB65**	42](#_Toc228559242)

[2.1.5. The synthesis of **BB66** ~ **BB68**	49](#_Toc228559243)

[2.2. Copies of NMR Spectra	52](#_Toc228559244)

# 1. Biology Experiments

## 1.1. General Methods

### 1.1.1. Treatment preparation

A stock solution of LPS (*Escherichia coli* O111:B4, Sigma-Aldrich, Cat. No. L4391) was prepared at 1 mg/mL in sterile, nuclease-free water (Ambion™, Invitrogen). Stock solutions of bilobalide analogues were prepared in dimethyl sulfoxide (DMSO; Sigma-Aldrich, Cat. No. D8418) at 50 mM. All stock solutions were stored at -20 °C until use. For experimental assays, compounds were diluted in culture medium or assay buffer to ensure the final DMSO concentration was lower than or equal to 0.1% (*v/v*).

### 1.1.2. Cytotoxicity

BV-2 cells were seeded in 96-well plates at 5 × 10^3^ cells per well and allowed to adhere overnight. Cells were then treated with bilobalide analogues (1.5–80 µM) for 24 h under standard culture conditions. Cell Counting Kit-8 (CCK-8) reagent was added according to the manufacturer’s instructions, and absorbance was measured at 450 nm. Viability data were normalized to DMSO vehicle controls.

### 1.1.3. Cell morphology evaluation

Morphological alterations associated with microglial activation were examined using bright-field microscopy. BV-2 cells were seeded in 6-well plates at a density of 2 × 10^5^ cells per well and incubated overnight. The following day, cells were treated with LPS (1 µg/mL) in the presence or absence of TAK-242 (10 µM; reference inhibitor) or bilobalide analogues (20 µM) for 24 h. Cell morphology was visualized using an inverted light microscope (ECLIPSE Ts2, Nikon), and representative images were documented to evaluate treatment-induced changes.

### 1.1.4. Quantitative real-time PCR (qPCR) analysis

Quantitative real-time PCR (qPCR) was performed by using TB Green® Premix Ex Taq™ (Til RNaseH Plus) (Takara Bio, Japan) on the Applied Biosystems QuantStudio 12K Flex Real-Time PCR System (Thermo Fisher Scientific, MA, US). Specific primer pairs used in this study are listed in **Table S1**.

**Table S1.** List of primers used in qPCR

| Gene | Forward primer sequence | Reverse primer sequence |
| --- | --- | --- |
| *Prep* | 5’-GCTATGGAAACTGTGCCTCG-3’ | 5’-GCTTTGTCCCGGGTAGTGAA-3’ |
| *Cox2* | 5’-GCGACATACTCAAGCAGGAGCA-3’ | 5’-AGTGGTAACCGCTCAGGTGTTG-3’ |
| *Il1b* | 5’-ACTGTGAAATGCCACCTTTTG-3’ | 5’-TGTTGATGTGCTGCTGTGAG-3’ |
| *Il6* | 5’-GCTGGTGACAACCACGGCCT-3’ | 5’-AGCCTCCGACTTGTGAAGTGGT-3’ |
| *Actb* | 5’-CATTGCTGACAGGATGCAGAAGG-3’ | 5’-TGCTGGAAGGTGGACAGTGAGG-3’ |

### 1.1.5. Western blotting

Equal amounts of protein were loaded onto FuturePAGE™ precast gels (ACE Biotechnology, China) and resolved using MOPS-SDS Running Buffer (ACE Biotechnology). PageRuler™ Prestained Protein Ladder (Thermo Fisher Scientific) was used as a molecular weight marker. Proteins were transferred onto PVDF membranes with a pore size of 0.2 μm (Thermo Scientific), followed by blocking with blocking buffer (ACE Biotechnology). Membranes were probed with the following primary antibodies, then the appropriate secondary antibodies (**Table S2**) were used. Protein bands were visualized using the Ultra High Sensitivity ECL Kit (MedChemExpress), for HRP-secondary antibodies, and imaged with the ChemiDoc MP Imaging System (Bio-Rad, CA, USA). Band intensities were quantified using ImageJ software (NIH, MD, USA).

**Table S2.** List of antibodies used in Western blot analysis

| Antibody | Cat. No. | Brand |
| --- | --- | --- |
| Anti-Prolyl Endopeptidase antibody | ab58988 | Abcam |
| Phospho-NF-κB p65 (Ser536) (93H1) Rabbit mAb | 3033 | Cell Signaling Technology |
| Phospho-p38 MAPK (Thr180/Tyr182) (D3F9) XP^®^ Rabbit mAb | 4511 | Cell Signaling Technology |
| Cox2 (D5H5) XP^®^ Rabbit mAb | 12282 | Cell Signaling Technology |
| NFκB p65 (F-6) Mouse mAb | sc-8008 | Santa Cruz Biotechnology |
| NF-kappaB p65 (D14E12) Rabbit mAb | 8242 | Cell Signaling Technology |
| p38 MAPK Rabbit mAb | 9212 | Cell Signaling Technology |
| PMPCA Polyclonal antibody | 26536-1-AP | Proteintech |
| GLUD1 Polyclonal antibody | 14299-1-AP | Proteintech |
| Stat3 (124H6) Mouse mAb | 9139 | Cell Signaling Technology |
| beta-Actin (8H10D10) Mouse mAb | 3700 | Cell Signaling Technology |
| Vinculin (7F9) Mouse mAb | sc-73614 | Santa Cruz Biotechnology |
| Anti-rabbit IgG, HRP-linked Antibody | 7074 | Cell Signaling Technology |
| Anti-mouse IgG, HRP-linked Antibody | 7076 | Cell Signaling Technology |
| Goat anti-Rabbit IgG (H+L) Secondary Antibody, DyLight™ 488 | 35552 | Invitrogen |
| Goat anti-Mouse IgG (H+L) Secondary Antibody, DyLight™ 488 | 35502 | Invitrogen |

### 1.1.6. Fluorescence imaging of neutrophil recruitment in zebrafish

Larvae were mounted dorsally in 1.2% low–melting-point agarose for imaging. Fluorescent images were acquired with a Nikon SMZ800N stereomicroscope equipped with a Touptek CCD camera at 8X magnification. Images were processed in Fiji (ImageJ), and neutrophils within the brain region were manually counted.

## 1.2. Supplementary Tables/ Figures

### 1.2.1. Cellular thermal shift assay–mass spectrometry (CETSA-MS) data

To elucidate the molecular target of **BB10**, we performed a cellular thermal shift assay coupled with mass spectrometry (CETSA-MS) in K562 chronic myeloid leukemia cells. **BB10**-induced thermal stabilization or destabilization of proteins was quantified and the significant hits are summarized in **Table S3**. PREP emerged as the top candidate, exhibiting pronounced thermal stabilization upon **BB10** treatment. These findings indicate that **BB10** engages PREP in living cells.

In contrast, PREP was not identified as a target of the parent compound **BB**, highlighting the importance of **BB10**-specific structural modifications for PREP binding. The CETSA-MS volcano plot for bilobalide treatment is shown in **Figure S1**, and the corresponding list of significantly affected proteins is provided in **Table S4**.

**Table S3.** List of significant protein targets of **BB10** from CETSA-MS analysis

| Uniprot ID | Gene/ Protein | Description | log_2_ Fold change  (BB10 vs DMSO) | -Log_10_ *P*-value |
| --- | --- | --- | --- | --- |
| Thermal stabilized proteins | | | | |
| P48147 | PREP | Prolyl endopeptidase | 0.7960 | 2.3604 |
| Q10713 | PMPCA | Mitochondrial-processing peptidase subunit alpha | 0.7528 | 2.1655 |
| P00367 | GLUD1 | Glutamate dehydrogenase 1, mitochondrial | 0.8282 | 1.9526 |
| P40763 | STAT3 | Signal transducer and activator of transcription 3 | 0.5380 | 1.9526 |
| Q8IWV2 | CNTN4 | Contactin-4 | 0.7209 | 1.5625 |
| Q9Y6V0 | PCLO | Protein piccolo | 0.7647 | 1.5499 |
| P50336 | PPOX | Protoporphyrinogen oxidase | 0.6210 | 1.5497 |
| O94864 | SUPT7L | STAGA complex 65 subunit gamma | 0.5257 | 1.5171 |
| P63173 | RPL38 | Large ribosomal subunit protein eL38 | 1.0079 | 1.4075 |
| O75449 | KATNA1 | Katanin p60 ATPase-containing subunit A1 | 0.6270 | 1.4075 |
| P50135 | HNMT | Histamine N-methyltransferase | 0.6609 | 1.3976 |
| Q8NBH2 | KY | Kyphoscoliosis peptidase | 0.5460 | 1.3918 |
| Q16854 | DGUOK | Deoxyguanosine kinase, mitochondrial | 0.6115 | 1.3808 |
| Q16718 | NDUFA5 | NADH dehydrogenase [ubiquinone] 1 alpha subcomplex subunit 5 | 0.5485 | 1.3159 |
| Thermal destabilized proteins | | | | |
| Q96KP4 | CNDP2 | Cytosolic non-specific dipeptidase | -0.5330 | 2.3604 |
| Q16822 | PCK2 | Phosphoenolpyruvate carboxykinase [GTP], mitochondrial | -0.5198 | 1.9526 |
| Q9Y663 | HS3ST3A1 | Heparan sulfate glucosamine 3-O-sulfotransferase 3A1 | -0.5517 | 1.9526 |
| P00505 | GOT2 | Aspartate aminotransferase, mitochondrial | -0.8245 | 1.9526 |
| P04040 | CAT | Catalase | -0.5504 | 1.7477 |
| Q14624 | ITIH4 | Inter-alpha-trypsin inhibitor heavy chain H4 | -0.5029 | 1.6912 |
| P04080 | CSTB | Cystatin-B | -0.7555 | 1.6459 |
| P15636 | Protease 1 | Protease 1 | -0.9155 | 1.6341 |
| P14174 | MIF | Macrophage migration inhibitory factor | -0.6367 | 1.5978 |
| Q9BV57 | ADI1 | Acireductone dioxygenase | -0.6905 | 1.5978 |
| Q7Z406 | MYH14 | Myosin-14 | -0.5321 | 1.5625 |
| Q8N7Z5 | ANKRD31 | Ankyrin repeat domain-containing protein 31 | -0.9293 | 1.5625 |
| P60673 | PFN3 | Profilin-3 | -0.7838 | 1.5509 |
| Q2LD37 | BLTP1 | Bridge-like lipid transfer protein family member 1 | -0.6471 | 1.5486 |
| Q9UM01 | SLC7A7 | Y+L amino acid transporter 1 | -0.5853 | 1.5171 |
| P17706 | PTPN2 | Tyrosine-protein phosphatase non-receptor type 2 | -0.7125 | 1.4267 |
| Q9UL42 | PNMA2 | Paraneoplastic antigen Ma2 | -0.5863 | 1.4229 |
| Q3ZBD7 | GPI | Glucose-6-phosphate isomerase | -0.5189 | 1.4075 |
| Q6P4F2 | FDX2 | Ferredoxin-2, mitochondrial | -0.5660 | 1.3809 |

**Figure S1.** Volcano plot of cellular thermal shift assay–mass spectrometry (CETSA-MS) data, comparing DMSO (0.1%) and bilobalide (**BB**) (20 µM) treatments.

**Table S4.** List of significant protein targets of bilobalide (**BB**) from CETSA-MS analysis

| Uniprot ID | Gene/ Protein | Description | log_2_ Fold change  (BB vs DMSO) | -Log_10_ *P*-value |
| --- | --- | --- | --- | --- |
| Thermal stabilized proteins | | | | |
| Q13526 | PIN1 | Peptidylprolyl cis/trans isomerase, NIMA-interacting 1 | 0.2835 | 1.5722 |
| Q00688 | FKBP3 | FKBP prolyl isomerase | 0.3116 | 1.5527 |
| Q9NTZ6 | RBM12 | RNA binding motif protein 12 | 0.3249 | 1.5503 |
| Q96BX8 | MOB3A | MOB kinase activator 3A | 0.3619 | 1.3781 |
| Q9NP77 | SSU72 | SSU72 homolog, RNA polymerase II CTD phosphatase | 0.4513 | 1.3781 |
| Q8N8S7 | ENAH | ENAH actin regulator | 0.3889 | 1.3565 |
| O75449 | KATNA1 | Katanin catalytic subunit A1 | 0.4518 | 1.3545 |
| Q53EL6 | PDCD4 | Programmed cell death 4 | 0.2886 | 1.3210 |
| Thermal destabilized proteins | | | | |
| P84243 | H3-3B | H3.3 histone A | -0.3414 | 1.5722 |
| Q14997 | PSME4 | Proteasome activator subunit 4 | -0.3379 | 1.5722 |
| P30154 | PPP2R1B | Protein phosphatase 2 scaffold subunit abeta | -0.3262 | 1.5722 |
| P51178 | PLCD1 | Phospholipase C delta 1 | -0.2757 | 1.5722 |
| P11413 | G6PD | Glucose-6-phosphate dehydrogenase | -0.3720 | 1.5503 |
| P55084 | HADHB | Hydroxyacyl-CoA dehydrogenase trifunctional multienzyme complex subunit beta | -0.3201 | 1.5503 |
| Q9UNH7 | SNX6 | Sorting nexin 6 | -0.3051 | 1.5503 |
| P35580 | MYH10 | Myosin heavy chain 10 | -0.3914 | 1.4326 |
| P39748 | FEN1 | Flap structure-specific endonuclease 1 | -0.3449 | 1.4326 |
| O95394 | PGM3 | Phosphoglucomutase 3 | -0.2935 | 1.4326 |
| Q58FG1 | HSP90AA4P | Heat shock protein 90 alpha family class A member 4, pseudogene | -0.2671 | 1.4326 |
| O00203 | AP3B1 | Adaptor related protein complex 3 subunit beta 1 | -0.5868 | 1.4155 |
| Q5TEC6 | H3-7 | H3.7 histone (putative) | -0.4801 | 1.3781 |
| Q6NZI2 | CAVIN1 | Caveolae associated protein 1 | -0.4471 | 1.3781 |
| P19623 | SRM | Spermidine synthase | -0.4222 | 1.3781 |
| P52298 | NCBP2 | Nuclear cap binding protein subunit 2 | -0.3982 | 1.3781 |
| P14324 | FDPS | Farnesyl diphosphate synthase | -0.3453 | 1.3781 |
| P04083 | ANXA1 | Annexin A1 | -0.3367 | 1.3781 |
| O75390 | CS | Citrate synthase | -0.3342 | 1.3781 |
| P04181 | OAT | Ornithine aminotransferase | -0.2806 | 1.3781 |
| P23434 | GCSH | Glycine cleavage system protein H | -0.2791 | 1.3781 |
| Q08J23 | NSUN2 | NOP2/Sun RNA methyltransferase 2 | -0.2730 | 1.3781 |
| O95149 | SNUPN | Snurportin 1 | -0.2786 | 1.3605 |
| P40939 | HADHA | Hydroxyacyl-CoA dehydrogenase trifunctional multienzyme complex subunit alpha | -0.3059 | 1.3565 |
| Q9H7Z7 | PTGES2 | Prostaglandin E synthase 2 | -0.2674 | 1.3330 |
| Q5T6V5 | QNG1 | Q-nucleotide N-glycosylase 1 | -0.4068 | 1.3308 |

### 1.2.2. Cellular thermal shift assay–Western blot (CETSA-WB) of **BB10** and identified targets from CETSA-MS

In addition to PREP (**Figure 2C**), we also examined several other highly enriched candidates identified by CETSA-MS (**Figure 2B** and **Table S3**). Specifically, the top four thermally stabilized proteins were validated using CETSA-WB analysis. As shown in **Figure S2**, **BB10** also altered the thermal stability of PMPCA, GLUD1, and STAT3, suggesting additional cellular engagement. The functional relevance of these proteins to the anti-neuroinflammatory phenotype remains to be determined.

**Figure S2.** CETSA-WB analysis of **A**) PMPCA, **B**) GLUD1, and **C**) STAT3 in LPS-stimulated BV-2 microglial cells treated with DMSO (0.1%) or **BB10** (50 µM). Band intensities of the proteins of interest (POIs) were quantified and normalized to β-actin, and the resulting POI/β-actin ratios are shown in the figures. **BB10** increased the thermal stability of PMPCA, GLUD1, and STAT3, suggesting target engagement in LPS-stimulated BV-2 cells. Data are presented as mean ± SD (n = 3). Statistical significance was analyzed using a paired *t*-test (*P* < 0.05).

### 1.2.3. Chemical structures of D-ring modification

**Figure S3** shows chemical structures of compounds tested in this study.

**Figure S3.** Chemical structures of D-ring modification

### 1.2.4. Screening of PREP inhibitory activity of bilobalide analogues

Bilobalide analogues were initially screened for PREP inhibitory activity at a concentration of 25 µM, with the percentage inhibition values summarized in **Table S5**.

**Table S5.** Screening of PREP inhibitory activity of bilobalide analogues

| Compounds | % Inhibition (mean ± SD) |
| --- | --- |
| BB | 15.9 ± 2.6 |
| BB01 | 21.1 ± 9.3 |
| BB03 | 12.4 ± 12.1 |
| BB10 | 83.3 ± 0.6 |
| BB43 | 9.3 ± 8.0 |
| BB44 | 0.4 ± 24.7 |
| BB45 | -8.2 ± 15.1 |
| BB48 | -10.6 ± 9.6 |
| BB49 | -4.1 ± 16.3 |
| BB50 | -4.2 ± 10.3 |
| BB51 | 26.6 ± 6.6 |
| BB53 | -16.4 ± 9.8 |
| BB46 | -11.7 ± 10.2 |
| BB47 | 22.6 ± 17.7 |
| BB52 | 1.7 ± 7.8 |
| BB54 | 28.4 ± 7.0 |
| BB56 | 82.0 ± 1.1 |

Data are presented as mean ± SD (n = 3).

### 1.2.5. Cytotoxicity of **BB10** and **BB56** against BV-2 microglia cells

To define non-cytotoxic concentration ranges for subsequent assays, we evaluated BV-2 cell viability following treatment with the test compounds. **BB10** exhibited no detectable cytotoxicity at concentrations up to 80 µM, maintaining >80% cell viability across all doses. Similarly, **BB56** was well tolerated up to 20 µM, with minimal loss of viability (<20%). However, higher concentrations (40–80 µM) of **BB56** reduced cell viability to approximately 73–78%, indicating dose-dependent toxicity (**Figure S4**). Based on these findings, concentrations ≤20 µM were selected for all anti-neuroinflammatory efficacy studies.

**Figure S4.** Cytotoxicity of BB10 and BB56 in BV-2 microglia cells. BV-2 cells were treated with **BB10** or **BB56** (1.25-80 µM) for 24 h. The cell viability was evaluated by CCK-8 assay, compared to the vehicle control. Data are presented as mean ± SD (n = 3).

### 1.2.6. LPS activates prolyl endopeptidase (PREP) protein expression

To assess how LPS-induced microglial activation influences PREP expression in BV-2 cells, we exposed the cells to increasing concentrations of LPS for 24 h. As shown in **Figure S5**, LPS treatment resulted in a dose-dependent increase in PREP expression, with significant upregulation detected at 1 and 2 µg/mL.

**Figure S5.** Microglial activation increases PREP expression. BV-2 cells were exposed to LPS at concentrations of 0.125-2 µg/mL for 24 h. Protein expression of PREP was determined by Western blot. Data are presented as mean ± SD (n = 3). Statistical analysis was performed using one-way ANOVA followed by Dunnett’s post hoc test. ***P* < 0.01, ****P* < 0.001.

### 1.2.7. Inhibition of PREP by KYP-2047 suppresses BV-2 microglial inflammation

To investigate whether PREP inhibition modulates neuroinflammation, we examined the effects of the selective PREP inhibitor KYP-2047. Treatment with KYP-2047 (10 and 20 µM) significantly reduced the mRNA expression of *Il1b* and *Cox2* in LPS-stimulated BV-2 cells. In contrast, *Il6* expression was not markedly affected by KYP-2047 (**Figure S6**).

**Figure S6.** Inhibition of PREP suppresses inflammation. The effect of KYP-2047 (1, 10, and 20 µM) on mRNA expression of pro-inflammatory genes *Il1b*, *Il6*, and *Cox2* in LPS-stimulated BV-2 cells. KYP-2047 significantly reduced *Il1b* and *Cox2* expression in LPS-stimulated BV-2 cells, whereas *Il6* was not significantly affected. Data are presented as mean ± SD (n = 9). Statistical analysis was performed using one-way ANOVA followed by Dunnett’s post hoc test. **P* < 0.05, ***P* < 0.01, ****P* < 0.001.

### 1.2.8. Dose-response curves of bilobalide analogues for PREP inhibitory activity

To quantify the inhibitory potency of bilobalide analogues against PREP, we assessed their activities using human recombinant PREP protein across a series of concentrations. Based on the initial screening results (**Figure 3B**), active compounds were further subjected to dose–response analysis to determine their half-maximal inhibitory concentrations (IC_50_). As shown in **Figure S7**, the bilobalide analogues exhibited IC_50_ values ranging from 6.9 to 22.6 µM. Notably, **BB56** and **BB60** demonstrated the most active compounds, with IC_50_ value of 9.6 and 6.9 µM, respectively.

**Figure S7.** Dose-response curves of bilobalide analogues for PREP inhibitory activity. Data are presented as mean ± SD (n = 3).

### 1.2.9. Quantitative microscale thermophoresis (MST) analysis of **BB10** and **BB56** binding to PREP mutants

To experimentally validate the predicted binding mode by molecular docking (**Figure 2H**), we generated a series of site-directed PREP mutants (PREP^F173A^, PREP^F476A, I478A^, and PREP^I591A, W595A^) targeting residues implicated in ligand interaction. Binding affinities of **BB10** and **BB56** toward these mutants were assessed using MST. Notably, **BB10** and **BB56** exhibited markedly reduced binding affinity across all mutant variants (**Figure S8**) compared to the wild-type protein (**Figure 2D**), indicating that these residues are critical for ligand engagement. These findings provide strong experimental support for the docking-predicted binding interface and reinforce the mechanistic basis of our structure-activity relationship (SAR) analysis.

**Figure S8.** MST analysis of **BB10** and **BB56** binding to PREP mutants. A series of site-directed PREP mutants (PREP^F173A^, PREP^F476A, I478A^, and PREP^I591A, W595A^) targeting residues implicated in ligand interaction. MST was used to determine the binding affinities of **BB10** and **BB56** toward recombinant PREP variants. Binding curves and derived dissociation constants (K_d_) are shown for **A-C**) **BB10** and **D-F**) **BB56**. Data are presented as mean ± SD from three independent experiments (n = 3).

### 1.2.10. Prep silencing in BV-2 cells by siRNA

To determine transfection efficiency, *Prep* expression was measured after siRNA-PREPs were transfected. The siRNA-PREP #1 and #2 efficiently reduced *Prep* mRNA levels compared to the negative control siRNA (siRNA-NC), as shown in **Figure S9**.

**Figure S9.** *Prep* silencing in BV-2 cells by siRNA. Two siRNA sequences (siRNA-PREP #1 and #2) effectively reduced *Prep* mRNA expression. Data are presented as mean ± SD (n = 9). Statistical analysis was performed using one-way ANOVA followed by Dunnett’s post hoc test. *****P* < 0.0001.

### 1.2.11. Effects of **BB56** on neutrophil recruitment in a zebrafish model of neuroinflammation

To explore whether **BB56** modulates neutrophil recruitment in an *in vivo* zebrafish neuroinflammation model, we first assessed immune cell infiltration in a zebrafish model of neuroinflammation. As shown in **Figure S10**, only a few neutrophils were detected in the brains of ddH_2_O-injected control larvae, whereas LPS injection induced a significant increase in neutrophil accumulation (n = 12 and 19 fish for control and LPS groups, respectively; *P* < 0.0001), confirming successful model induction. Under these conditions, BB56 and KYP-2047 did not significantly reduce LPS-induced neutrophil recruitment (**BB56**: n = 15 fish, *P* = 0.2504; KYP-2047: n = 12 fish, *P* = 0.8615), suggesting that this acute neutrophil recruitment readout may not fully capture the PREP-dependent anti-inflammatory effects observed in BV-2 cells.

**Figure S10.** Effects of **BB56** on neutrophil recruitment in a zebrafish model of neuroinflammation. **A**) Representative fluorescence images of neutrophil recruitment in Tg(mpx:EGFP) zebrafish. The 5 dpf zebrafish larvae were ventricular injected with LPS (5 mg/mL) or ddH_2_O and immediately treated with **BB56** (50 μM) or KYP-2047 (100 μM). At 24 hours post injection (hpi), larvae were randomly picked for imaging using a Nikon SMZ800N stereomicroscope equipped with a Touptek CCD camera at 8X magnification **B**) Number of neutrophils within the brain region. Data are presented as mean ± SD (n = 12-19). Statistical analysis was performed using one-way ANOVA followed by Dunnett’s post hoc test. *****P* < 0.0001. ns = not significant.

### 1.2.12. Original Western blots

Original Western blot images from all three biological replicates used for protein expression quantification are shown in **Figures S11-S22**.

**Figure S11.** Original Western blot images from the three biological experiments shown in **Figure 1I**.

**Figure S12.** Original Western blot images from the three biological experiments shown in **Figure 2B**.

**Figure S13.** Original Western blot images from the three biological experiments shown in **Figure 3D**.

**Figure S14.** Original Western blot images from the three biological experiments shown in **Figure 4H**.

**Figure S15.** Original Western blot images from the three biological experiments shown in **Figure 5A**.

**Figure S16.** Original Western blot images from the three biological experiments shown in **Figure 5D**.

**Figure S17.** Original Western blot images from the three biological experiments shown in **Figure 5F**.

**Figure S18A.** Original Western blot images from the three biological experiments shown in **Figure 5G**; p-NF-κB p65 (Ser536), total NF-κB, and vinculin, experiment #1.

**Figure S18B.** Original Western blot images from the three biological experiments shown in **Figure 5G**; p-NF-κB p65 (Ser536), total NF-κB, and vinculin, experiment #2.

**Figure S18C.** Original Western blot images from the three biological experiments shown in **Figure 5G**; p-NF-κB p65 (Ser536), total NF-κB, and vinculin, experiment #3.

**Figure S18D.** Original Western blot images from the three biological experiments shown in **Figure 5G**; p-p38 MAPK (Thr180/Tyr182), total p38 MAPK, and vinculin, experiment #1.

**Figure S18E.** Original Western blot images from the three biological experiments shown in **Figure 5G**; p-p38 MAPK (Thr180/Tyr182), total p38 MAPK, and vinculin, experiment #2.

**Figure S18F.** Original Western blot images from the three biological experiments shown in **Figure 5G**; p-p38 MAPK (Thr180/Tyr182), total p38 MAPK, and vinculin, experiment #3.

**Figure S19.** Original Western blot images from the three biological experiments shown in **Figure S5**.

**Figure S20.** Original Western blot images from the three biological experiments shown in **Figure S2A**.

**Figure S21.** Original Western blot images from the three biological experiments shown in **Figure S2B**.

**Figure S22.** Original Western blot images from the three biological experiments shown in **Figure S2C**.

# 2. Chemical Experiments

## 2.1. Chemical Synthesis

### 2.1.1. General Chemical Experimental Information

Unless otherwise specified, syntheses and manipulations of air- and moisture-sensitive materials were carried out under nitrogen atmosphere using standard Schlenk techniques. All glassware was oven-dried immediately prior to use. Authentic (–)-bilobalide (CAS: 33570-04-6) is commercially available and can be obtained from established chemical suppliers, including TCI, Sigma-Aldrich, MedChemExpress, Fluorochem or Energy Chemicals. Other materials were purchased from commercial sources and used without additional purification unless otherwise noted. Reactions were magnetically stirred and monitored by analytical thin-layer chromatography (TLC). TLC was performed on Merck silica gel 60 F_254_ TLC glass plates and observed by exposure to ultraviolet light.

On Chemical Reagent silica gel (200-300 mesh), silica chromatography was done using the appropriate solvent system. The purity of the resulting compounds was determined by Agilent 6430 triple quadrupole liquid chromatography/tandem mass spectrometry (LC-MS/MS) (Agilent Technologies, Santa Clara, CA, United States) and Agilent 1260 Infinity II high-performance liquid chromatography (HPLC) system (Agilent Technologies, Santa Clara, CA, United States). Nuclear magnetic resonance (NMR) spectra of ^1^H and ^13^C were obtained using a Bruker Ultrashield 400 Plus NMR spectrometer or Bruker Ascend 700 NMR spectrometer. Chemical shifts (δ) were indicated in parts per million (ppm). Tetramethylsilane was used as an internal reference. Coupling constants, *J*, were expressed in Hertz. The coupling constants were expressed in the following descriptions, including singlet, s; doublet, d; triplet, t; quartet, q; multiplet, m; broad, br. Samples were tested using flow-injection analysis into a Thermo Q Exactive^TM^ Focus Hybrid Quadrupole-Orbitrap^TM^ Mass Spectrometer for high-resolution mass spectrometry (HRMS) analysis.

### 2.1.2. General method A: synthesis of diBz-Bilobalide analogues **BB37** to **BB42**, **M-10** to **M-12**

To an oven-dried flask containing a magnetic stirrer bar was added bilobalide (0.10 g, 0.31 mmol, 1.0 equiv.), followed by the addition of anhydrous dichloromethane (DCM, 3.0 mL) at 0 ℃. Then benzoyl chloride analogues (0.93 mmol, 3.0 equiv.) and tetramethylethylenediamine (TMEDA, 136 µL, 0.93 mmol, 3.0 equiv.) were added. The reaction was stirred under nitrogen atmosphere at room temperature for 3.0 h. Upon completion, the reaction solution was diluted with DCM and transferred to a separatory funnel. The organic layer was washed with saturated ammonium chloride aqueous solution (NH_4_Cl) and subsequently washed with saturated sodium bicarbonate aqueous solution (NaHCO_3_). After that, the organic layer was washed with brine, dried over anhydrous MgSO_4_, filtered and concentrated in *vacuo*. The crude product was then purified by silica gel column chromatography with elution system (hexane/EtOAc/DCM) to afford **BB37** to **BB42**, **M-10** to **M-12**.

**(2*R*,3a'*S,*4*R*,6'*R*,7a'*S*)-6'-(*tert*-butyl)-2',4',5-trioxohexahydro-4'*H*,6'*H*-spiro[furan-3,8'-[3a,6]methanofuro[3,2-*c*]pyran]-2,4-diyl bis(2-(trifluoromethyl)benzoate) (BB37)**

Using **method A** with 2-(trifluoromethyl)benzoyl chloride (**M-1**, 137 µL, 0.93 mmol, 3.0 equiv.); yield 90% (279 µmol, 187 mg); white powder. Purification by column chromatography (SiO_2_, hexane/EtOAc/DCM= 5:1:1→ 3:1:1→ 2:1:1, v/v).

R*_f_* = 0.62 (hexane/EtOAc = 1:1). ^1^H NMR (700 MHz, CDCl_3_) δ [ppm] = 7.83 (dd, *J* = 7.7, 3.8 Hz, 2H), 7.72 (d, *J* = 7.6 Hz, 2H), 7.70 – 7.67 (m, 2H), 7.67 – 7.63 (m, 1H), 7.62 (t, *J* = 7.6 Hz, 1H), 7.22 (s, 1H), 6.19 (s, 1H), 4.40 (dd, *J* = 7.9, 4.4 Hz, 1H), 2.95 (d, *J* = 18.2 Hz, 1H), 2.86 (d, *J* = 6.7 Hz, 1H), 2.83 – 2.80 (m, 1H), 2.77 (dd, *J* = 15.2, 4.4 Hz, 1H), 1.28 (s, 9H). ^13^C{^1^H} NMR (176 MHz, CDCl_3_) δ [ppm] = 173.3, 169.1, 167.7, 164.1, 163.7, 133.1, 132.32, 132.27, 132.2, 131.9, 129.2, 128.7, 127.8, 127.2, 127.1, 100.8, 94.1, 78.3, 67.6, 63.6, 62.9, 35.9, 35.0, 31.7, 26.6. ^19^F{^1^H} NMR (471 MHz, CDCl_3_) δ [ppm] = -59.04, -59.30. HRMS (ESI) m/z: [M + Na]^+^ Calcd for C_31_H_24_F_6_O_10_Na^+^ 693.1166; found 693.1154.

**(2*R*,3a'*S*,4*R*,6'*R*,7a'*S*)-6'-(*tert*-butyl)-2',4',5-trioxohexahydro-4'*H*,6'*H*-spiro[furan-3,8'-[3a,6]methanofuro[3,2-*c*]pyran]-2,4-diyl bis(4-fluorobenzoate)** **(BB38)**

Using **method A** with 4-fluorobenzoyl chloride (**M-2**, 110 µL, 0.93 mmol, 3.0 equiv.); yield 77% (239 µmol, 137 mg); white powder. Purification by column chromatography (SiO_2_, hexane/EtOAc/DCM= 4:1:1→ 2:1:1, v/v).

R*_f_* = 0.59 (hexane/EtOAc = 3:1). ^1^H NMR (700 MHz, CDCl_3_) δ [ppm] = 8.09 – 8.06 (m, 2H), 8.04 – 8.01 (m, 2H), 7.34 (s, 1H), 7.27 (s, 1H), 7.25 (d, *J* = 8.4 Hz, 1H), 7.18 (t, *J* = 8.5 Hz, 2H), 6.23 (s, 1H), 4.47 (dd, *J* = 7.5, 4.6 Hz, 1H), 3.27 (d, *J* = 17.9 Hz, 1H), 3.12 (d, *J* = 17.8 Hz, 1H), 2.87 – 2.82 (m, 2H), 1.27 (s, 9H). ^13^C{^1^H} NMR (176 MHz, CDCl_3_) δ [ppm] = 173.2, 169.0, 168.8, 167.5, 166.0, 162.9, 162.8, 132.9, 132.8, 124.3, 123.9, 116.8, 116.7, 116.3, 116.1, 101.0, 94.3, 78.7, 67.5, 63.9, 62.7, 35.8, 34.9, 32.4, 26.6. ^19^F{^1^H} NMR (471 MHz, CDCl_3_) δ [ppm] = -101.22, -102.08. HRMS (ESI) m/z: [M + Na]^+^ Calcd for C_29_H_24_F_2_O_10_Na^+^ 593.1230; found 593.1229.

**(2*R*,3a'*S*,4*R*,6'*R*,7a'*S*)-6'-(*tert*-butyl)-2',4',5-trioxohexahydro-4'*H*,6'*H*-spiro[furan-3,8'-[3a,6]methanofuro[3,2-*c*]pyran]-2,4-diyl bis(4-(trifluoromethyl)benzoate)** **(BB39)**

Using **method A** with 4-(trifluoromethyl)benzoyl chloride (**M-3**, 138 µL, 0.93 mmol, 3.0 equiv.); yield 29% (89.9 µmol, 60.3 mg); white powder. Purification by column chromatography (SiO_2_, hexane/EtOAc/DCM= 5:1:1→ 4:1:1→ 3:1:1, v/v).

R*_f_* = 0.63 (hexane/EtOAc = 2:1). ^1^H NMR (400 MHz, CDCl_3_) δ [ppm] = 8.16 (d, *J* = 8.1 Hz, 2H), 8.10 (d, *J* = 8.1 Hz, 2H), 7.86 (d, *J* = 8.1 Hz, 2H), 7.77 (d, *J* = 8.2 Hz, 2H), 7.37 (s, 1H), 6.28 (s, 1H), 4.49 (t, *J* = 6.1 Hz, 1H), 3.25 (d, *J* = 18.0 Hz, 1H), 3.13 (d, *J* = 18.0 Hz, 1H), 2.86 (d, *J* = 6.1 Hz, 2H), 1.29 (s, 9H). ^13^C{^1^H} NMR (176 MHz, CDCl_3_) δ [ppm] = 173.1, 168.7, 168.6, 162.84, 162.79, 136.5, 131.2, 130.9, 130.6, 130.4, 126.4, 125.9, 125.7, 124.2, 122.6, 101.0, 94.6, 78.6, 67.6, 63.8, 62.7, 35.9, 34.9, 32.4, 26.6. ^19^F{^1^H} NMR (471 MHz, CDCl_3_) δ [ppm] = -63.30, -63.35. HRMS (ESI) m/z: [M + Na]^+^ Calcd for C_31_H_24_F_6_O_10_Na^+^ 693.1166; found 693.1156.

**(2*R*,3a'*S*,4*R*,6'*R*,7a'*S*)-6'-(*tert*-butyl)-2',4',5-trioxohexahydro-4'*H*,6'*H*-spiro[furan-3,8'-[3a,6]methanofuro[3,2-*c*]pyran]-2,4-diyl bis(3-(trifluoromethyl)benzoate)** **(BB40)**

Using **method A** with 3-(trifluoromethyl)benzoyl chloride (**M-4**, 140 µL, 0.93 mmol, 3.0 equiv.); yield 96% (298 µmol, 200 mg); white powder. Purification by column chromatography (SiO_2_, hexane/EtOAc/DCM= 1:1:1, v/v).

R*_f_* = 0.6 (hexane/EtOAc = 2:1). ^1^H NMR (700 MHz, CDCl_3_) δ [ppm] = 8.29 (s, 2H), 8.24 (d, *J* = 8.0 Hz, 1H), 8.18 (d, *J* = 7.9 Hz, 1H), 7.95 (dd, *J* = 13.7, 7.9 Hz, 2H), 7.71 (dt, *J* = 22.3, 7.9 Hz, 2H), 7.36 (d, *J* = 1.9 Hz, 1H), 6.37 (s, 1H), 4.49 (dt, *J* = 7.5, 2.8 Hz, 1H), 3.32 (dd, *J* = 18.5, 1.8 Hz, 1H), 3.24 (dd, *J* = 18.4, 1.8 Hz, 1H), 2.96 (dd, *J* = 17.3, 2.5 Hz, 1H), 2.69 – 2.64 (m, 1H), 1.34 (s, 9H). ^13^C{^1^H} NMR (176 MHz, CDCl_3_) δ [ppm] = 171.8, 168.6, 168.5, 162.9, 162.7, 133.3, 132.9, 131.8, 131.5, 131.2, 130.1, 130.0, 128.5, 128.2, 127.5, 126.8, 124.1, 101.6, 93.4, 79.1, 64.3, 62.7, 61.4, 35.5, 35.2, 30.3, 27.0. ^19^F{^1^H} NMR (471 MHz, CDCl_3_) δ [ppm] = -62.92, -62.99. HRMS (ESI) m/z: [M + Na]^+^ Calcd for C_31_H_24_F_6_O_10_Na^+^ 693.1166; found 693.1157.

**(2*R*,3a'*S*,4*R*,6'*R*,7a'*S*)-6'-(*tert*-butyl)-2',4',5-trioxohexahydro-4'*H*,6'*H*-spiro[furan-3,8'-[3a,6]methanofuro[3,2-*c*]pyran]-2,4-diyl bis(2-fluorobenzoate)** **(BB41)**

Using **method A** with 2-fluorobenzoyl chloride (**M-5**, 111 µL, 0.93 mmol, 3.0 equiv.); yield 86% (267 µmol, 152 mg); white powder. Purification by column chromatography (SiO_2_, hexane/EtOAc/DCM= 4:1:1→ 2:1:1, v/v).

R*_f_* = 0.6 (hexane/EtOAc = 2:1). ^1^H NMR (700 MHz, CDCl_3_) δ [ppm] = 8.01 (t, *J* = 7.5 Hz, 2H), 7.70 – 7.61 (m, 2H), 7.33 (d, *J* = 8.6 Hz, 2H), 7.28 (d, *J* = 7.9 Hz, 1H), 7.24 (d, *J* = 8.7 Hz, 1H), 7.20 – 7.16 (m, 1H), 6.25 (s, 1H), 4.44 (t, *J* = 6.1 Hz, 1H), 3.56 (d, *J* = 18.1 Hz, 1H), 3.01 (d, *J* = 18.1 Hz, 1H), 2.82 (d, *J* = 6.1 Hz, 2H), 1.27 (s, 9H). ^13^C{^1^H} NMR (176 MHz, CDCl_3_) δ [ppm] = 173.8, 169.0, 163.2, 162.9, 161.8, 161.4, 136.5, 136.4, 133.1, 132.8, 124.8, 124.4, 117.5, 117.2, 116.4, 116.2, 100.9, 94.2, 78.5, 67.1, 63.6, 62.7, 35.8, 34.8, 31.6, 26.6. ^19^F{^1^H} NMR (471 MHz, CDCl_3_) δ [ppm] = -106.82, -107.01. HRMS (ESI) m/z: [M + Na]^+^ Calcd for C_29_H_24_F_2_O_10_Na^+^ 593.1230; found 593.1228.

**(2*R*,3a'*S*,4*R*,6'*R*,7a'*S*)-6'-(*tert*-butyl)-2',4',5-trioxohexahydro-4'*H*,6'*H*-spiro[furan-3,8'-[3a,6]methanofuro[3,2-*c*]pyran]-2,4-diyl bis(3-fluorobenzoate) (BB42)**

Using **method A** with 3-fluorobenzoyl chloride (**M-6**, 113 µL, 0.93 mmol, 3.0 equiv.); yield 76% (236 µmol, 134 mg); white powder. Purification by column chromatography (SiO_2_, hexane/EtOAc/DCM= 4:1:1→ 3:1:1, v/v).

R*_f_* = 0.75 (hexane/EtOAc = 2:1). ^1^H NMR (700 MHz, CDCl_3_) δ [ppm] = 7.85 (d, *J* = 7.8 Hz, 1H), 7.78 (d, *J* = 7.8 Hz, 1H), 7.72 – 7.68 (m, 2H), 7.54 (dq, *J* = 20.8, 6.4 Hz, 2H), 7.40 (dt, *J* = 15.9, 8.1 Hz, 2H), 7.33 (s, 1H), 6.33 (d, *J* = 1.8 Hz, 1H), 4.49 – 4.46 (m, 1H), 3.29 (d, *J* = 20.4 Hz, 1H), 3.21 (d, *J* = 18.7 Hz, 1H), 2.99 (d, *J* = 15.1 Hz, 1H), 2.66 (dd, *J* = 14.8, 8.1 Hz, 1H), 1.33 (s, 9H). ^13^C{^1^H} NMR (176 MHz, CDCl_3_) δ [ppm] = 171.9, 168.7, 168.6, 163.6, 163.0, 162.7, 162.1, 131.2, 129.7, 129.3, 125.9, 122.2, 121.9, 117.4, 117.3, 117.1, 117.0, 101.6, 93.2, 79.1, 64.2, 62.7, 61.4, 35.5, 35.2, 30.3, 27.0. ^19^F{^1^H} NMR (471 MHz, CDCl_3_) δ [ppm] = -110.05, -111.14. HRMS (ESI) m/z: [M + Na]^+^ Calcd for C_29_H_24_F_2_O_10_Na^+^ 593.1230; found 593.1223.

**(2*R*,3a'*S*,4*R*,6'*R*,7a'*S*)-6'-(*tert*-butyl)-2',4',5-trioxohexahydro-4'*H*,6'*H*-spiro[furan-3,8'-[3a,6]methanofuro[3,2-*c*]pyran]-2,4-diyl bis(4-methylbenzoate)** **(M-10)**

Using **method A** with p-toluoyl chloride (**M-7**, 123 µL, 0.93 mmol, 3.0 equiv.); yield 94% (291 µmol, 164 mg); white powder. Purification by column chromatography (SiO_2_, hexane/EtOAc/DCM= 5:1:1→ 4:1:1, v/v).

R*_f_* = 0.57 (hexane/EtOAc = 2:1). ^1^H NMR (700 MHz, CDCl_3_) δ [ppm] = 7.97 (d, *J* = 8.2 Hz, 2H), 7.92 (d, *J* = 8.2 Hz, 2H), 7.36 (d, *J* = 8.0 Hz, 2H), 7.35 (s, 1H), 7.31 (d, *J* = 8.0 Hz, 2H), 6.22 (s, 1H), 4.44 (dd, *J* = 7.9, 4.3 Hz, 1H), 3.36 (d, *J* = 17.9 Hz, 1H), 3.07 (d, *J* = 17.9 Hz, 1H), 2.86 (dd, *J* = 15.2, 4.3 Hz, 1H), 2.81 (dd, *J* = 15.2, 7.9 Hz, 1H), 2.50 (s, 3H), 2.46 (s, 3H), 1.26 (s, 9H). ^13^C{^1^H} NMR (176 MHz, CDCl_3_) δ [ppm] = 173.2, 169.3, 169.0, 163.7, 146.2, 145.7, 130.3, 130.2, 129.9, 129.5, 125.3, 124.9, 100.9, 94.1, 78.6, 67.4, 63.9, 62.7, 35.8, 34.8, 32.3, 26.5, 22.1.

**(2*R*,3a'*S*,4*R*,6'*R*,7a'*S*)-6'-(*tert*-butyl)-2',4',5-trioxohexahydro-4'*H*,6'*H*-spiro[furan-3,8'-[3a,6]methanofuro[3,2-*c*]pyran]-2,4-diyl bis(4-ethylbenzoate) (M-11)**

Using **method A** with 4-ethylbenzoyl chloride (**M-8**, 138 µL, 0.93 mmol, 3.0 equiv.); yield 87% (270 µmol, 159 mg); white powder. Purification by column chromatography (SiO_2_, hexane/EtOAc/DCM= 4:1:1→ 3:1:1, v/v).

R*_f_* = 0.68 (hexane/EtOAc = 2:1). ^1^H NMR (700 MHz, CDCl_3_) δ [ppm] = 8.00 (d, *J* = 8.2 Hz, 2H), 7.96 (d, *J* = 8.3 Hz, 2H), 7.39 (d, *J* = 8.1 Hz, 2H), 7.33 (d, *J* = 8.1 Hz, 2H), 7.30 (d, *J* = 8.0 Hz, 1H), 6.23 (s, 1H), 4.45 (dd, *J* = 7.9, 4.3 Hz, 1H), 3.38 (d, *J* = 17.9 Hz, 1H), 3.08 (d, *J* = 17.9 Hz, 1H), 2.87 (dd, *J* = 15.3, 4.3 Hz, 1H), 2.83 – 2.80 (m, 1H), 2.78 (dd, *J* = 14.3, 6.6 Hz, 2H), 2.76 – 2.73 (m, 2H), 1.32 (t, *J* = 7.6 Hz, 3H), 1.30 – 1.28 (m, 3H), 1.27 (s, 9H). ^13^C{^1^H} NMR (176 MHz, CDCl_3_) δ [ppm] = 173.3, 169.3, 169.0, 163.7, 152.3, 151.7, 150.8, 130.5, 130.4, 128.8, 128.3, 128.2, 125.5, 125.1, 100.9, 94.1, 78.7, 67.4, 63.9, 62.7, 35.8, 34.9, 32.4, 29.3, 29.2, 26.5, 15.3, 15.1.

**(2*R*,3a'*S*,4*R*,6'*R*,7a'*S*)-6'-(*tert*-butyl)-2',4',5-trioxohexahydro-4'*H*,6'*H*-spiro[furan-3,8'-[3a,6]methanofuro[3,2-*c*]pyran]-2,4-diyl bis(4-(dimethylamino)benzoate) (M-12)**

Using **method A** with 4-(dimethylamino)benzoyl chloride (**M-9**, 141 µL, 0.93 mmol, 3.0 equiv.); yield 63% (195 µmol, 121 mg); white powder. Purification by column chromatography (SiO_2_, hexane/EtOAc/DCM= 4:1:1→ 3:1:1→ 2:1:1, v/v).

R*_f_* = 0.40 (hexane/EtOAc = 1:1). ^1^H NMR (700 MHz, CDCl_3_) δ [ppm] = 7.93 (d, *J* = 9.0 Hz, 2H), 7.90 (d, *J* = 8.9 Hz, 2H), 7.34 (s, 1H), 6.70 (d, *J* = 9.0 Hz, 2H), 6.66 (d, *J* = 9.0 Hz, 2H), 6.23 (s, 1H), 4.42 (dd, *J* = 8.0, 4.2 Hz, 1H), 3.44 (d, *J* = 17.9 Hz, 1H), 3.11 (s, 6H), 3.10 (s, 1H), 3.07 (s, 6H), 2.87 (dd, *J* = 15.1, 4.2 Hz, 1H), 2.77 (dd, *J* = 15.1, 8.0 Hz, 1H), 1.26 (s, 9H). ^13^C{^1^H} NMR (176 MHz, CDCl_3_) δ [ppm] = 173.5, 170.0, 169.4, 163.9, 163.8, 154.3, 154.2, 132.3, 132.2, 114.2, 113.5, 111.1, 110.7, 100.9, 93.8, 78.7, 66.9, 64.0, 62.7, 40.2, 40.1, 35.7, 34.9, 32.3, 26.6.

### 2.1.3. General method B: synthesis of D-ring analogues **BB43** to **BB54**

To an oven-dried round bottom flask with a magnetic stir bar was added **1** (50 mg, 93.5 µmol, 1.0 equiv.) or **BB38** (50 mg, 87.6 µmol, 1.0 equiv.), followed by the addition of tetrahydrofuran (THF, 1 mL). Substituted amines **M-13** to **M-21** (1.5 equiv.) and triethylamine (TEA, 3.0 equiv.) were then added to the flask. The resulting solution was stirred at room temperature for 3 h. Upon completion indicated by TLC, the solution was concentrated under reduced pressure, and redissolved in DCM. The organic layer was washed with saturated NH_4_Cl and subsequently washed with brine, dried over anhydrous MgSO_4_, filtered and concentrated in *vacuo*. The crude product was purified via column chromatography using Hexane/EtOAc provide the corresponding products **BB43** to **BB54**.

**(3a*S*,5a*S*,8*R*,9*R*,10a*S*)-9-(*tert*-butyl)-9-hydroxy-6-(4-methoxybenzyl)-2,4,7-trioxooctahydro-4*H*,9*H*-furo[3'',2'':2',3']cyclopenta[1',2':3,4]furo[2,3-*b*]pyrrol-8-yl benzoate (BB43)**

Using **method B** with compound **1** (50 mg, 93.5 µmol, 1.0 equiv.), 4-methoxybenzylamine (**M-13**, 18.3 µL, 140 µmol, 1.5 equiv.) and TEA (39.2 µL, 281 µmol, 3.0 equiv.); yield 87% (81.3 µmol, 45 mg); white powder. Purification by column chromatography (SiO_2_, hexane/EtOAc= 4:1→ 2:1, v/v).

R*_f_* = 0.51 (hexane: EtOAc = 1:1). ^1^H NMR (700 MHz, CDCl_3_) δ [ppm] = 8.00 (d, *J* = 7.7 Hz, 2H), 7.63 (t, *J* = 7.5 Hz, 1H), 7.50 (t, *J* = 7.7 Hz, 2H), 7.25 (s, 1H), 6.89 (d, *J* = 8.2 Hz, 2H), 6.31 (s, 1H), 5.69 (s, 1H), 5.19 (t, *J* = 7.1 Hz, 1H), 4.94 (d, *J* = 14.5 Hz, 1H), 4.12 (d, *J* = 14.5 Hz, 1H), 3.81 (s, 3H), 3.49 (s, 1H), 3.16 (d, *J* = 19.0 Hz, 1H), 2.85 (d, *J* = 19.0 Hz, 1H), 2.59 (dd, *J* = 14.0, 7.2 Hz, 1H), 2.22 (dd, *J* = 14.0, 7.1 Hz, 1H), 0.98 (s, 9H). ^13^C{^1^H} NMR (176 MHz, CDCl_3_) δ [ppm] = 177.9, 173.6, 166.6, 165.4, 159.8, 134.4, 130.32, 130.26, 128.9, 127.9, 126.1, 114.5, 87.7, 86.7, 83.2, 71.0, 62.3, 59.5, 55.4, 44.1, 43.3, 37.3, 36.5, 26.4. HRMS (ESI) m/z: [M + Na]^+^ Calcd for C_30_H_31_NO_9_Na^+^ 572.1891; found 572.1885.

**(3a*S*,5a*S*,8*R*,9*R*,10a*S*)-9-(*tert*-butyl)-9-hydroxy-6-neopentyl-2,4,7-trioxooctahydro-4*H*,9*H*-furo[3'',2'':2',3']cyclopenta[1',2':3,4]furo[2,3-*b*]pyrrol-8-yl benzoate (BB44)**

Using **method B** with compound **1** (50 mg, 93.5 µmol, 1.0 equiv.), 2,2-dimethyl propylamine (**M-14**, 16.4 µL, 140 µmol, 1.5 equiv.) and TEA (39.2 µL, 281 µmol, 3.0 equiv.); yield 96% (89.8 µmol, 45 mg); white powder. Purification by column chromatography (SiO_2_, hexane/EtOAc= 3:1, v/v).

R*_f_* = 0.63 (hexane/EtOAc = 2:1). ^1^H NMR (700 MHz, CDCl_3_) δ [ppm] = 7.99 (dd, *J* = 8.3, 1.4 Hz, 2H), 7.62 (t, *J* = 7.3 Hz, 1H), 7.49 (t, *J* = 7.9 Hz, 2H), 6.37 (s, 1H), 6.12 (s, 1H), 5.21 (t, *J* = 7.1 Hz, 1H), 3.60 (d, *J* = 14.2 Hz, 1H), 3.17 (d, *J* = 18.9 Hz, 1H), 2.94 (d, *J* = 14.2 Hz, 1H), 2.86 (d, *J* = 19.0 Hz, 1H), 2.63 (dd, *J* = 14.1, 7.1 Hz, 1H), 2.27 (dd, *J* = 14.0, 7.1 Hz, 1H), 1.13 (s, 9H), 1.00 (s, 9H). ^13^C{^1^H} NMR (176 MHz, CDCl_3_) δ [ppm] = 177.9, 173.5, 167.9, 165.4, 134.4, 130.2, 128.9, 127.9, 88.9, 87.6, 83.2, 70.6, 62.6, 59.3, 50.9, 43.4, 37.5, 36.5, 34.1, 28.5, 26.5. HRMS (ESI) m/z: [M + Na]^+^ Calcd for C_27_H_33_NO_8_Na^+^ 522.2098; found 522.2092

**(3a*S*,5a*S*,8*R*,9*R*,10a*S*)-9-(*tert*-butyl)-6-(cyclohexylmethyl)-9-hydroxy-2,4,7-trioxooctahydro-4*H*,9*H*-furo[3'',2'':2',3']cyclopenta[1',2':3,4]furo[2,3-*b*]pyrrol-8-yl benzoate** **(BB45)**

Using **method B** with compound **1** (50 mg, 93.5 µmol, 1.0 equiv.), cyclohexanemethylamine (**M-15**, 18.2 µL, 140 µmol, 1.5 equiv.) and TEA (39.2 µL, 281 µmol, 3.0 equiv.); yield 91% (85.1 µmol, 45 mg); white powder. Purification by column chromatography (SiO_2_, hexane/EtOAc= 2:1, v/v).

R*_f_* = 0.59 (hexane/EtOAc = 2:1). ^1^H NMR (700 MHz, CDCl_3_) δ [ppm] = 7.99 (d, *J* = 8.5 Hz, 2H), 7.62 (t, *J* = 7.5 Hz, 1H), 7.49 (t, *J* = 7.9 Hz, 2H), 6.36 (s, 1H), 5.93 (s, 1H), 5.21 (t, *J* = 7.2 Hz, 1H), 3.46 (dd, *J* = 14.0, 8.4 Hz, 1H), 3.19 (d, *J* = 18.8 Hz, 1H), 3.07 (dd, *J* = 14.0, 6.7 Hz, 1H), 2.88 (d, *J* = 19.0 Hz, 1H), 2.62 (dd, *J* = 14.1, 7.2 Hz, 1H), 2.26 (dd, *J* = 14.1, 7.2 Hz, 1H), 1.94 (s, 1H), 1.77 – 1.73 (m, 2H), 1.67 (d, *J* = 12.7 Hz, 1H), 1.65 – 1.59 (m, 2H), 1.25 (d, *J* = 7.1 Hz, 2H), 1.21 (dd, *J* = 12.4, 2.6 Hz, 1H), 1.18 (d, *J* = 13.1 Hz, 1H), 1.11 (s, 9H), 1.01 (dd, *J* = 12.4, 9.3 Hz, 1H), 0.98 – 0.93 (m, 1H). ^13^C{^1^H} NMR (176 MHz, CDCl_3_) δ [ppm] = 178.0, 173.5, 167.2, 165.4, 134.4, 130.2, 128.9, 127.9, 88.2, 87.7, 83.2, 70.8, 62.5, 59.4, 47.0, 43.4, 37.5, 36.6, 35.6, 31.0, 30.9, 26.3, 25.6. HRMS (ESI) m/z: [M + Na]^+^ Calcd for C_29_H_35_NO_8_Na^+^ 548.2255; found 548.2250.

**(3a*S*,5a*S*,8*R*,9*R*,10a*S*)-6-benzyl-9-(*tert*-butyl)-9-hydroxy-2,4,7-trioxooctahydro-4*H*,9*H*-furo[3'',2'':2',3']cyclopenta[1',2':3,4]furo[2,3-*b*]pyrrol-8-yl 4-fluorobenzoate** **(BB46)**

Using **method B** with **BB38** (50 mg, 87.6 µmol, 1.0 equiv.), benzylamine (**M-16**, 14.3 µL, 131 µmol, 1.5 equiv.) and TEA (36.7 µL, 263 µmol, 3.0 equiv.); yield 89% (78.0 µmol, 41.9 mg); white powder. Purification by column chromatography (SiO_2_, hexane/EtOAc= 2:1, v/v).

R*_f_* = 0.48 (hexane/EtOAc = 2:1). ^1^H NMR (700 MHz, CDCl_3_) δ [ppm] = 8.04 – 8.01 (m, 2H), 7.38 (t, *J* = 7.3 Hz, 2H), 7.35 – 7.33 (m, 1H), 7.32 (d, *J* = 7.1 Hz, 2H), 7.17 (t, *J* = 8.5 Hz, 2H), 6.32 (s, 1H), 5.69 (s, 1H), 5.19 (t, *J* = 7.1 Hz, 1H), 5.00 (d, *J* = 14.5 Hz, 1H), 4.18 (d, *J* = 14.6 Hz, 1H), 3.14 (d, *J* = 19.0 Hz, 1H), 2.87 (d, *J* = 19.0 Hz, 1H), 2.59 (dd, *J* = 14.0, 7.2 Hz, 1H), 2.19 (dd, *J* = 14.0, 7.1 Hz, 1H), 1.84 (s, 1H), 0.98 (s, 9H). ^13^C{^1^H} NMR (176 MHz, CDCl_3_) δ [ppm] = 177.7, 173.6, 167.4, 166.6, 166.0, 164.5, 134.2, 133.1 (d), 129.3, 128.8, 124.1, 116.3 (d), 87.7, 86.7, 83.2, 71.1, 62.3, 59.5, 44.7, 43.4, 37.3, 36.6, 26.3. ^19^F{^1^H} NMR (471 MHz, CDCl_3_) δ [ppm] = -102.92. HRMS (ESI) m/z: [M + Na]^+^ Calcd for C_29_H_28_FNO_8_Na^+^ 560.1691; found 560.1690.

**(3aS,5aS,8R,9R,10aS)-9-(tert-butyl)-9-hydroxy-6-(4-methoxybenzyl)-2,4,7-trioxooctahydro-4H,9H-furo[3'',2'':2',3']cyclopenta[1',2':3,4]furo[2,3-b]pyrrol-8-yl 4-fluorobenzoate** **(BB47)**

Using **method B** with **BB38** (50 mg, 87.6 µmol, 1.0 equiv.), 4-methoxybenzylamine (**M-13**, 17.1 µL, 131 µmol, 1.5 equiv.) and TEA (36.7 µL, 263 µmol, 3.0 equiv.); yield 91% (79.7 µmol, 45.2 mg); white powder. Purification by column chromatography (SiO_2_, hexane/EtOAc= 2:1, v/v).

R*_f_* = 0.35 (hexane/EtOAc = 2:1). ^1^H NMR (700 MHz, CDCl_3_) δ 8.02 (dd, *J* = 8.5, 5.2 Hz, 2H), 7.25 (d, *J* = 9.3 Hz, 2H), 7.17 (t, *J* = 8.4 Hz, 2H), 6.89 (d, *J* = 8.2 Hz, 2H), 6.29 (s, 1H), 5.69 (s, 1H), 5.18 (t, *J* = 7.1 Hz, 1H), 4.93 (d, *J* = 14.5 Hz, 1H), 4.12 (d, *J* = 14.5 Hz, 1H), 3.81 (s, 3H), 3.12 (d, *J* = 19.0 Hz, 1H), 2.85 (d, *J* = 19.0 Hz, 1H), 2.60 (dd, *J* = 14.1, 7.2 Hz, 1H), 2.18 (dd, *J* = 14.0, 7.0 Hz, 1H), 2.00 (s, 1H), 0.98 (s, 9H).^13^C{^1^H} NMR (176 MHz, CDCl_3_) δ [ppm] = 177.8, 173.6, 166.5, 165.9, 164.5, 159.8, 133.0, 130.3, 126.1, 124.1, 116.2, 114.5, 87.6, 86.8, 83.2, 71.2, 62.3, 59.5, 55.4, 44.2, 43.3, 37.3, 36.6, 26.4. ^19^F{^1^H} NMR (471 MHz, CDCl_3_) δ [ppm] = -102.95. HRMS (ESI) m/z: [M + Na]^+^ Calcd for C_30_H_30_FNO_9_Na^+^ 590.1797; found 590.1798.

**(3a*S*,5a*S*,8*R*,9*R*,10a*S*)-9-(*tert*-butyl)-9-hydroxy-6-(3-hydroxy-4-methoxyphenethyl)-2,4,7-trioxooctahydro-4*H*,9*H*-furo[3'',2'':2',3']cyclopenta[1',2':3,4]furo[2,3-*b*]pyrrol-8-yl benzoate** **(BB48)**

Using **method B** with compound **1** (50 mg, 93.5 µmol, 1.0 equiv.), 3-hydroxy-4-methoxyphenethylamine (**M-17**, 23.4 mg, 140 µmol, 1.5 equiv.) and TEA (39.2 µL, 281 µmol, 3.0 equiv.); yield 46% (43.0 µmol, 24.9 mg); white powder. Purification by column chromatography (SiO_2_, hexane/EtOAc= 2:1, v/v).

R*_f_* = 0.37 (hexane/EtOAc = 1:1). ^1^H NMR (700 MHz, CDCl_3_) δ [ppm] = 7.97 (d, *J* = 7.8 Hz, 2H), 7.61 (t, *J* = 7.3 Hz, 1H), 7.49 (t, *J* = 7.5 Hz, 2H), 6.82 – 6.79 (m, 2H), 6.72 (d, *J* = 8.2 Hz, 1H), 6.16 (s, 1H), 5.61 (s, 1H), 5.16 (t, *J* = 7.0 Hz, 1H), 3.87 (s, 3H), 3.86 (d, *J* = 6.6 Hz, 1H), 3.50 (dt, *J* = 14.6, 7.5 Hz, 1H), 3.06 (d, *J* = 19.0 Hz, 1H), 2.96 (dt, *J* = 14.8, 7.7 Hz, 1H), 2.85 (dt, *J* = 13.5, 6.2 Hz, 1H), 2.77 (d, *J* = 19.1 Hz, 1H), 2.57 (dd, *J* = 13.7, 7.4 Hz, 1H), 2.19 (dd, *J* = 13.9, 7.0 Hz, 1H), 0.95 (s, 9H). ^13^C{^1^H} NMR (176 MHz, CDCl_3_) δ [ppm] = 178.0, 173.6, 167.1, 165.4, 146.0, 145.7, 134.4, 130.7, 130.2, 128.9, 127.9, 120.2, 115.1, 111.2, 88.3, 87.6, 83.2, 70.8, 62.6, 59.4, 56.2, 43.2, 42.0, 37.3, 36.4, 32.4, 26.3. HRMS (ESI) m/z: [M + Na]^+^ Calcd for C_31_H_33_NO_10_Na^+^ 602.1997; found 602.1984.

**(3a*S*,5a*S*,8*R*,9*R*,10a*S*)-6-(2-(benzo[*d*][1,3]dioxol-5-yl)ethyl)-9-(*tert*-butyl)-9-hydroxy-2,4,7-trioxooctahydro-4*H*,9*H*-furo[3'',2'':2',3']cyclopenta[1',2':3,4]furo[2,3-*b*]pyrrol-8-yl benzoate (BB49)**

Using **method B** with compound **1** (50 mg, 93.5 µmol, 1.0 equiv.), homopiperonylamine (**M-18**, 19.2 µL, 140 µmol, 1.5 equiv.) and TEA (39.2 µL, 281 µmol, 3.0 equiv.); yield 82% (76.7 µmol, 44.3 mg); white powder. Purification by column chromatography (SiO_2_, hexane/EtOAc= 2:1, v/v).

R*_f_* = 0.55 (hexane/EtOAc = 1:1). ^1^H NMR (700 MHz, CDCl_3_) δ [ppm] = 7.97 (dd, *J* = 8.3, 1.4 Hz, 2H), 7.62 (t, *J* = 7.4 Hz, 1H), 7.49 (t, *J* = 7.9 Hz, 2H), 6.76 (d, *J* = 7.8 Hz, 1H), 6.73 – 6.67 (m, 2H), 6.18 (s, 1H), 5.94 (s, 2H), 5.63 (s, 1H), 5.30 (s, 1H), 5.16 (t, *J* = 7.1 Hz, 1H), 3.85 (ddd, *J* = 14.2, 7.1, 5.7 Hz, 1H), 3.52 – 3.48 (m, 1H), 3.07 (d, *J* = 19.0 Hz, 1H), 2.97 (dt, *J* = 14.9, 7.6 Hz, 1H), 2.88 – 2.84 (m, 1H), 2.78 (d, *J* = 19.1 Hz, 1H), 2.58 (dd, *J* = 14.1, 7.2 Hz, 1H), 2.20 (dd, *J* = 14.1, 7.1 Hz, 1H), 0.97 (s, 9H). ^13^C{^1^H} NMR (176 MHz, CDCl_3_) δ [ppm] = 177.9, 173.5, 167.1, 165.4, 148.2, 146.7, 134.4, 131.3, 130.2, 128.9, 127.9, 121.8, 109.2, 108.8, 101.2, 88.3, 87.6, 83.2, 70.8, 62.7, 59.4, 43.3, 42.2, 37.4, 36.4, 32.9, 26.4. HRMS (ESI) m/z: [M + Na]^+^ Calcd for C_31_H_31_NO_10_Na^+^ 600.1840; found 600.1831.

**(3a*S*,5a*S*,8*R*,9*R*,10a*S*)-9-(*tert*-butyl)-9-hydroxy-6-(4-nitrophenethyl)-2,4,7-trioxooctahydro-4*H*,9*H*-furo[3'',2'':2',3']cyclopenta[1',2':3,4]furo[2,3-*b*]pyrrol-8-yl benzoate (BB50)**

Using **method B** with compound **1** (50 mg, 93.5 µmol, 1.0 equiv.), 4-nitrophenethylamine hydrochloride (**M-19**, 28.4 mg, 140 µmol, 1.5 equiv.) and TEA (52.1 µL, 374 µmol, 4.0 equiv.); yield 44% (41.1 µmol, 23.8 mg); white powder. Purification by column chromatography (SiO_2_, hexane/EtOAc= 1:1, v/v).

R*_f_* = 0.65 (hexane/EtOAc = 1:1). ^1^H NMR (700 MHz, CDCl_3_) δ [ppm] = 8.21 (d, *J* = 8.6 Hz, 2H), 7.97 (d, *J* = 7.2 Hz, 2H), 7.63 (t, *J* = 7.5 Hz, 1H), 7.49 (t, *J* = 7.7 Hz, 2H), 7.44 (d, *J* = 8.4 Hz, 2H), 6.20 (s, 1H), 5.67 (s, 1H), 5.17 (t, *J* = 7.1 Hz, 1H), 3.88 (ddd, *J* = 14.0, 7.9, 5.7 Hz, 1H), 3.65 (dt, *J* = 14.7, 7.7 Hz, 1H), 3.18 (dt, *J* = 15.1, 7.9 Hz, 1H), 3.08 – 3.03 (m, 2H), 2.75 (d, *J* = 18.9 Hz, 1H), 2.59 (dd, *J* = 14.1, 7.2 Hz, 1H), 2.22 (dd, *J* = 14.2, 7.1 Hz, 1H), 1.91 (s, 1H), 0.98 (s, 9H). ^13^C{^1^H} NMR (176 MHz, CDCl_3_) δ [ppm] = 177.6, 173.2, 167.2, 165.3, 147.3, 145.2, 134.5, 130.2, 129.8, 129.0, 127.7, 124.3, 88.1, 87.7, 83.1, 70.6, 62.6, 59.3, 43.3, 41.7, 37.4, 36.4, 33.4, 26.3. HRMS (ESI) m/z: [M + Na]^+^ Calcd for C_30_H_30_N_2_O_10_Na^+^ 601.1793; found 601.1784.

**(3a*S*,5a*S*,8*R*,8a*S*,9*R*,10a*S*)-9-(*tert*-butyl)-9-hydroxy-6-(4-hydroxyphenethyl)-2,4,7-trioxooctahydro-4*H*,9*H*-furo[3'',2'':2',3']cyclopenta[1',2':3,4]furo[2,3-*b*]pyrrol-8-yl benzoate (BB51)**

Using **method B** with compound **1** (50 mg, 93.5 µmol, 1.0 equiv.), tyramine (**M-20**, 19.2 mg, 140 µmol, 1.5 equiv.) and TEA (39.2 µL, 281 µmol, 3.0 equiv.); yield 90% (84.2 µmol, 46.3 mg); white powder. Purification by column chromatography (SiO_2_, hexane/EtOAc= 4:1→1:1, v/v).

R*_f_* = 0.31 (hexane/EtOAc = 1:1). ^1^H NMR (700 MHz, Methanol-d_4_) δ [ppm] = 7.91 (d, *J* = 8.4 Hz, 2H), 7.62 (t, *J* = 7.3 Hz, 1H), 7.45 (t, *J* = 7.9 Hz, 2H), 7.05 (d, *J* = 8.5 Hz, 2H), 6.70 (d, *J* = 8.6 Hz, 2H), 6.11 (s, 1H), 5.65 (s, 1H), 5.06 (t, *J* = 7.1 Hz, 1H), 3.78 (dt, *J* = 13.2, 6.3 Hz, 1H), 3.53 (ddd, *J* = 14.5, 8.2, 6.5 Hz, 1H), 2.91 (dt, *J* = 14.8, 7.5 Hz, 1H), 2.86 – 2.80 (m, 1H), 2.77 (d, *J* = 10.9 Hz, 2H), 2.62 (dd, *J* = 13.8, 7.1 Hz, 1H), 2.00 (dd, *J* = 13.8, 7.2 Hz, 1H), 0.87 (s, 9H). ^13^C{^1^H} NMR (176 MHz, CDCl_3_) δ [ppm] = 179.4, 175.8, 169.3, 166.5, 157.5, 135.3, 131.0, 130.9, 129.9, 129.5, 128.2, 116.6, 89.9, 87.4, 85.4, 72.3, 64.4, 60.6, 43.1, 43.0, 38.3, 37.1, 33.0, 26.9. HRMS (ESI) m/z: [M + Na]^+^ Calcd for C_30_H_31_NO_9_Na^+^, 572.1891; found, 572.1885.

**(3a*S*,5a*S*,8*R*,9*R*,10a*S*)-9-(*tert*-butyl)-9-hydroxy-6-(4-hydroxyphenethyl)-2,4,7-trioxooctahydro-4*H*,9*H*-furo[3'',2'':2',3']cyclopenta[1',2':3,4]furo[2,3-b]pyrrol-8-yl 4-fluorobenzoate (BB52)**

Using **method B** with **BB38** (50 mg, 87.6 µmol, 1.0 equiv.), tyramine (**M-20**, 18.0 mg, 131 µmol, 1.5 equiv.) and TEA (36.7 µL, 263 µmol, 3.0 equiv.); yield 89% (78.0 µmol, 44.3 mg); white powder. Purification by column chromatography (SiO_2_, hexane/EtOAc= 4:1, v/v).

R*_f_* = 0.52 (hexane/EtOAc = 1:1). ^1^H NMR (700 MHz, Methonal-d_4_) δ [ppm] = 8.04 – 8.00 (m, 2H), 7.23 (t, *J* = 8.7 Hz, 2H), 7.09 (d, *J* = 7.9 Hz, 2H), 6.76 – 6.72 (m, 2H), 6.15 (s, 1H), 5.69 (s, 1H), 5.11 (t, *J* = 7.1 Hz, 1H), 3.82 (dt, *J* = 13.3, 6.4 Hz, 1H), 3.57 (dt, *J* = 14.7, 7.2 Hz, 1H), 2.95 (dt, *J* = 14.9, 7.5 Hz, 1H), 2.88 (dt, *J* = 13.6, 6.3 Hz, 1H), 2.83 – 2.79 (m, 2H), 2.66 (dd, *J* = 13.8, 7.2 Hz, 1H), 2.04 (dd, *J* = 13.8, 7.1 Hz, 1H), 0.91 (s, 9H). ^13^C{^1^H} NMR (176 MHz, Methonal-d_4_) δ [ppm] = 179.3, 175.8, 169.2, 167.1, 165.5, 157.5, 133.95, 133.89, 130.9, 129.7, 125.9, 117.0, 116.9, 116.6, 89.8, 87.4, 85.4, 72.5, 64.4, 60.6, 43.1, 43.0, 38.3, 37.2, 33.0, 26.9. ^19^F{^1^H} NMR (471 MHz, MeOD) δ [ppm] = -105.93. HRMS (ESI) m/z: [M + Na]^+^ Calcd for C_30_H_30_FNO_9_Na^+^ 590.1797; found 590.1797.

**(3a*S*,5a*S*,8*R*,8a*S*,9*R*,10a*S*)-9-(*tert*-butyl)-9-hydroxy-6-(4-methoxyphenethyl)-2,4,7-trioxooctahydro-4*H*,9*H*-furo[3'',2'':2',3']cyclopenta[1',2':3,4]furo[2,3-*b*]pyrrol-8-yl benzoate (BB53)**

Using **method B** with compound **1** (50 mg, 93.5 µmol, 1.0 equiv.), 4-methoxyphenethylamine (**M-21**, 20.5 µL, 140 µmol, 1.5 equiv.) and TEA (39.2 µL, 281 µmol, 3.0 equiv.); yield 83% (77.6 µmol, 43.7 mg); white powder. Purification by column chromatography (SiO_2_, hexane/EtOAc= 2:1, v/v).

R*_f_* = 0.30 (hexane/EtOAc = 2:1). ^1^H NMR (700 MHz, CDCl_3_) δ [ppm] = 7.97 (d, *J* = 5.5 Hz, 2H), 7.61 (d, *J* = 6.1 Hz, 1H), 7.49 (d, *J* = 6.0 Hz, 2H), 7.15 (d, *J* = 5.4 Hz, 2H), 6.85 (d, *J* = 5.9 Hz, 2H), 6.15 (s, 1H), 5.61 (s, 1H), 5.18 – 5.11 (m, 1H), 3.91 – 3.84 (m, 1H), 3.79 (s, 3H), 3.53 (t, *J* = 7.0 Hz, 1H), 3.01 (t, *J* = 13.7 Hz, 2H), 2.90 (d, *J* = 6.4 Hz, 1H), 2.72 (d, *J* = 20.0 Hz, 1H), 2.57 (d, *J* = 7.2 Hz, 1H), 2.17 (d, *J* = 9.1 Hz, 1H), 0.93 (s, 9H). ^13^C{^1^H} NMR (176 MHz, CDCl_3_) δ [ppm] = 178.1, 173.6, 167.1, 165.4, 158.7, 134.4, 130.2, 129.8, 129.4, 128.9, 127.9, 114.4, 88.3, 87.5, 83.2, 70.8, 62.7, 59.4, 55.5, 43.1, 42.0, 37.3, 36.3, 32.2, 26.4. HRMS (ESI) m/z: [M + Na]^+^ Calcd for C_31_H_33_NO_9_Na^+^ 586.2048; found 586.2040.

**(3a*S*,5a*S*,8*R*,9*R*,10a*S*)-9-(*tert*-butyl)-9-hydroxy-6-(4-methoxyphenethyl)-2,4,7-trioxooctahydro-4*H*,9*H*-furo[3'',2'':2',3']cyclopenta[1',2':3,4]furo[2,3-*b*]pyrrol-8-yl 4-fluorobenzoate (BB54)**

Using **method B** with **BB38** (50 mg, 87.6 µmol, 1.0 equiv.), 4-methoxyphenethylamine (**M-21**, 19.2 µL, 131 µmol, 1.5 equiv.) and TEA (36.7 µL, 263 µmol, 3.0 equiv.); yield 88% (77.1 µmol, 44.8 mg); white powder. Purification by column chromatography (SiO_2_, hexane/EtOAc= 2:1, v/v).

R*_f_* = 0.34 (hexane/EtOAc = 2:1). ^1^H NMR (700 MHz, CDCl_3_) δ [ppm] = 8.00 (dd, *J* = 9.0, 5.2 Hz, 2H), 7.16 (t, *J* = 8.2 Hz, 4H), 6.88 – 6.84 (m, 2H), 6.14 (s, 1H), 5.58 (s, 1H), 5.16 (t, *J* = 7.1 Hz, 1H), 3.88 (ddd, *J* = 14.3, 7.1, 5.7 Hz, 1H), 3.79 (s, 3H), 3.53 (ddd, *J* = 14.5, 8.2, 6.7 Hz, 1H), 3.03 – 2.97 (m, 2H), 2.90 (dt, *J* = 14.5, 6.3 Hz, 1H), 2.74 (d, *J* = 19.0 Hz, 1H), 2.56 (dd, *J* = 14.1, 7.2 Hz, 1H), 2.16 (dd, *J* = 14.1, 7.0 Hz, 1H), 1.80 (s, 1H), 0.94 (s, 9H). ^13^C{^1^H} NMR (176 MHz, CDCl_3_) δ [ppm] = 177.7, 173.6, 167.0, 165.9, 164.4, 158.8, 133.02, 132.96, 129.8, 129.3, 124.1, 116.3, 116.2, 114.5, 88.2, 87.7, 83.1, 70.9, 62.6, 59.4, 55.5, 43.4, 41.9, 37.3, 36.3, 32.2, 26.3. ^19^F{^1^H} NMR (471 MHz, CDCl_3_) δ [ppm] = -103.04. HRMS (ESI) m/z: [M + Na]^+^ Calcd for C_31_H_32_FNO_9_Na^+^ 604.1953; found 604.1956.

### 2.1.4. General method C: synthesis of **BB55** to **BB60**, **BB63** to **BB65**

To an oven-dried round bottom flask with a magnetic stirbar was added **BB37-BB42**, **M-10-M-12** (0.14 mmol, 1.0 equiv.), followed by the addition of acetonitrile (ACN, 2 mL). Dopamine hydrochloride **M-22** (29.2 mg, 154 µmol, 1.1 equiv.) and TEA (273 µL, 1.96 mmol, 14.0 equiv.) were then added to the flask. The resulting solution was stirred at room temperature for 3 h. Upon completion indicated by TLC, the solution was concentrated under reduced pressure, diluted with saturated NH_4_Cl solution. The residue was dissolved with DCM and the organic layer was washed with brine. The combined organic layers were dried over MgSO_4_, filtered and concentrated in *vacuo*. The crude product was purified via column chromatography using DCM/EtOAc provide the corresponding products **BB55** to **BB60**, **BB63** to **BB65**.

**(3a*S*,5a*S*,8*R*,9*R*,10a*S*)-9-(*tert*-butyl)-6-(3,4-dihydroxyphenethyl)-9-hydroxy-2,4,7-trioxooctahydro-4*H*,9*H*-furo[3'',2'':2',3']cyclopenta[1',2':3,4]furo[2,3-*b*]pyrrol-8-yl 2-(trifluoromethyl)benzoate (BB55)**

Using **method C** with **BB37** (93.9 mg, 0.14 mmol, 1.0 equiv.); yield 19% (26.6 µmol, 16.9 mg); white powder. Purification by column chromatography (SiO_2_, DCM/EtOAc= 1:1, v/v).

R*_f_* = 0.65 (DCM/EtOAc = 1:1). ^1^H NMR (700 MHz, CDCl_3_) δ [ppm] = 7.75 (dd, *J* = 19.3, 7.2 Hz, 2H), 7.71 – 7.64 (m, 2H), 6.81 (d, *J* = 8.0 Hz, 1H), 6.72 (d, *J* = 2.0 Hz, 1H), 6.64 (dd, *J* = 8.0, 2.0 Hz, 1H), 6.13 (s, 1H), 5.55 (s, 1H), 5.06 (t, *J* = 7.0 Hz, 1H), 3.96 (dt, *J* = 13.5, 6.4 Hz, 1H), 3.53 – 3.49 (m, 1H), 2.99 (dt, *J* = 14.7, 7.2 Hz, 1H), 2.88 (q, *J* = 7.8, 7.1 Hz, 1H), 2.85 (d, *J* = 18.5 Hz, 1H), 2.71 (d, *J* = 18.5 Hz, 1H), 2.52 (dd, *J* = 14.2, 7.2 Hz, 1H), 2.07 (dd, *J* = 14.2, 6.8 Hz, 1H), 0.93 (s, 9H). ^13^C{^1^H} NMR (176 MHz, CDCl_3_) δ [ppm] = 177.6, 172.7, 167.3, 164.8, 144.1, 143.5, 132.5, 132.2, 130.4, 129.3, 127.0, 121.2, 115.7, 115.3, 88.2, 87.6, 82.9, 71.1, 62.4, 59.6, 43.2, 41.5, 37.2, 36.9, 31.8, 26.2. ^19^F{^1^H} NMR (471 MHz, CDCl_3_) δ [ppm] = -59.16. HRMS (ESI) m/z: [M + Na]^+^ Calcd for C_31_H_30_F_3_NO_10_Na^+^ 656.1714; found 656.1713.

**(3a*S*,5a*S*,8*R*,9*R*,10a*S*)-9-(*tert*-butyl)-6-(3,4-dihydroxyphenethyl)-9-hydroxy-2,4,7-trioxooctahydro-4*H*,9*H*-furo[3'',2'':2',3']cyclopenta[1',2':3,4]furo[2,3-b]pyrrol-8-yl 4-fluorobenzoate** **(BB56)**

Using **method C** with **BB38** (79.9 mg, 0.14 mmol, 1.0 equiv.); yield 59% (82.6 µmol, 48.2 mg); white powder. Purification by column chromatography (SiO_2_, DCM/EtOAc= 4:1→2:1, v/v).

R*_f_* = 0.55 (DCM/EtOAc = 2:1). ^1^H NMR (700 MHz, CD_3_OD) δ [ppm] = 8.02 (dd, *J* = 8.9, 5.2 Hz, 2H), 7.24 (t, *J* = 8.7 Hz, 2H), 6.72 (d, *J* = 8.0 Hz, 1H), 6.69 (d, *J* = 2.1 Hz, 1H), 6.58 (dd, *J* = 8.1, 2.1 Hz, 1H), 6.17 (s, 1H), 5.64 (s, 1H), 5.11 (t, *J* = 7.1 Hz, 1H), 3.82 (dt, *J* = 14.1, 6.1 Hz, 1H), 3.53 (ddd, *J* = 14.5, 8.6, 6.3 Hz, 1H), 2.90 – 2.87 (m, 1H), 2.85 (d, *J* = 9.3 Hz, 2H), 2.82 (dd, *J* = 8.4, 5.9 Hz, 1H), 2.66 (dd, *J* = 13.8, 7.2 Hz, 1H), 2.04 (dd, *J* = 13.8, 7.1 Hz, 1H), 0.91 (s, 9H). ^13^C{^1^H} NMR (176 MHz, CD_3_OD) δ [ppm] = 179.3, 175.9, 169.2, 165.5, 146.7, 145.3, 133.94, 133.89, 130.5, 121.0, 117.0, 116.9, 116.8, 116.7, 89.9, 87.4, 85.4, 72.5, 64.4, 60.6, 43.1, 43.0, 38.3, 37.3, 33.3, 26.9. ^19^F{^1^H} NMR (471 MHz, MeOD) δ [ppm] = -105.93. HRMS (ESI) m/z: [M + Na]^+^ Calcd for C_30_H_30_FNO_10_Na^+^ 606.1746; found 606.1745.

**(3a*S*,5a*S*,8*R*,9*R*,10a*S*)-9-(*tert*-butyl)-6-(3,4-dihydroxyphenethyl)-9-hydroxy-2,4,7-trioxooctahydro-4*H*,9*H*-furo[3'',2'':2',3']cyclopenta[1',2':3,4]furo[2,3-*b*]pyrrol-8-yl 4-methylbenzoate (BB57)**

Using **method C** with **M-10** (78.8 mg, 0.14 mmol, 1.0 equiv.); yield 65% (91.0 µmol, 52.7 mg); white powder. Purification by column chromatography (SiO_2_, DCM/EtOAc= 6:1→5:1→2:1, v/v).

R*_f_* = 0.64 (DCM/EtOAc = 1:1). ^1^H NMR (700 MHz, CDCl_3_) δ [ppm] = 7.83 (d, *J* = 8.2 Hz, 2H), 7.28 – 7.26 (m, 2H), 6.79 (d, *J* = 8.0 Hz, 1H), 6.73 (d, *J* = 2.0 Hz, 1H), 6.62 (dd, *J* = 8.2, 2.0 Hz, 1H), 6.05 (s, 1H), 5.56 (s, 1H), 5.14 (t, *J* = 7.1 Hz, 1H), 3.89 (dt, *J* = 13.4, 6.3 Hz, 1H), 3.50 (ddd, *J* = 14.0, 8.0, 6.2 Hz, 1H), 2.98 (d, *J* = 19.2 Hz, 1H), 2.95 (dd, *J* = 15.2, 7.9 Hz, 1H), 2.85 (dt, *J* = 14.7, 6.3 Hz, 1H), 2.73 (d, *J* = 19.1 Hz, 1H), 2.55 (dd, *J* = 14.0, 7.1 Hz, 1H), 2.39 (s, 3H), 2.13 (dd, *J* = 14.0, 7.1 Hz, 1H), 0.86 (s, 9H). ^13^C{^1^H} NMR (176 MHz, CDCl_3_) δ [ppm] = 178.2, 174.0, 167.7, 165.7, 145.7, 144.0, 143.5, 130.2, 129.7, 129.5, 124.9, 121.2, 115.8, 115.4, 88.4, 87.6, 83.4, 70.8, 62.6, 59.4, 43.0, 41.6, 37.2, 36.3, 31.9, 26.2, 21.9. HRMS (ESI) m/z: [M + Na]^+^ Calcd for C_31_H_33_NO_10_Na^+^ 602.1997; found 602.1997.

**(3a*S*,5a*S*,8*R*,9*R*,10a*S*)-9-(*tert*-butyl)-6-(3,4-dihydroxyphenethyl)-9-hydroxy-2,4,7-trioxooctahydro-4*H*,9*H*-furo[3'',2'':2',3']cyclopenta[1',2':3,4]furo[2,3-*b*]pyrrol-8-yl 4-ethylbenzoate (BB58)**

Using **method C** with **M-11** (82.7 mg, 0.14 mmol, 1.0 equiv.); yield 76% (106 µmol, 63.2 mg); white powder. Purification by column chromatography (SiO_2_, DCM/EtOAc= 5:1→3:1, v/v).

R*_f_* = 0.43 (DCM/EtOAc = 2:1). ^1^H NMR (700 MHz, CDCl_3_) δ [ppm] = 7.86 (d, *J* = 8.2 Hz, 2H), 7.30 (d, *J* = 7.8 Hz, 2H), 6.79 (d, *J* = 8.2 Hz, 1H), 6.73 (s, 1H), 6.63 (d, *J* = 8.1 Hz, 1H), 6.06 (s, 1H), 5.55 (s, 1H), 5.15 (t, *J* = 7.0 Hz, 1H), 3.90 (dt, *J* = 13.4, 6.4 Hz, 1H), 3.49 (dt, *J* = 14.4, 7.2 Hz, 1H), 3.00 (d, *J* = 19.0 Hz, 1H), 2.96 (dd, *J* = 15.0, 7.8 Hz, 1H), 2.85 (dt, *J* = 13.6, 6.2 Hz, 1H), 2.74 (d, *J* = 19.2 Hz, 1H), 2.69 (q, *J* = 7.5 Hz, 2H), 2.55 (dd, *J* = 14.1, 7.2 Hz, 1H), 2.13 (dd, *J* = 14.0, 7.2 Hz, 1H), 1.27 – 1.22 (m, 3H), 0.86 (s, 9H). ^13^C{^1^H} NMR (176 MHz, CDCl_3_) δ [ppm] = 178.2, 174.0, 167.7, 165.7, 151.8, 144.0, 143.5, 130.4, 128.5, 128.2, 125.1, 121.2, 115.7, 115.4, 88.4, 87.6, 83.4, 70.8, 62.6, 59.4, 43.0, 41.5, 37.2, 36.3, 31.8, 29.2, 15.2. HRMS (ESI) m/z: [M + Na]^+^ Calcd for C_32_H_35_NO_10_Na^+^ 616.2153; found 616.2158.

**(3a*S*,5a*S*,8*R*,9*R*,10a*S*)-9-(*tert*-butyl)-6-(3,4-dihydroxyphenethyl)-9-hydroxy-2,4,7-trioxooctahydro-4*H*,9*H*-furo[3'',2'':2',3']cyclopenta[1',2':3,4]furo[2,3-*b*]pyrrol-8-yl 4-(dimethylamino)benzoate (BB59)**

Using **method C** with **M-12** (86.9 mg, 0.14 mmol, 1.0 equiv.); yield 96% (134 µmol, 81.8 mg); white powder. Purification by column chromatography (SiO_2_, DCM/EtOAc= 5:1→2:1, v/v).

R*_f_* = 0.25 (DCM/EtOAc = 2:1). ^1^H NMR (700 MHz, CDCl_3_) δ [ppm] = 7.81 (dd, *J* = 9.0, 1.9 Hz, 2H), 6.80 (dd, *J* = 8.0, 3.5 Hz, 1H), 6.73 (s, 1H), 6.66 – 6.61 (m, 3H), 6.01 – 5.98 (m, 1H), 5.51 (d, *J* = 2.2 Hz, 1H), 5.14 (t, *J* = 7.1 Hz, 1H), 3.94 (ddd, *J* = 13.9, 7.8, 5.7 Hz, 1H), 3.49 (s, 1H), 3.45 (dt, *J* = 11.8, 4.1 Hz, 1H), 3.08 (d, *J* = 2.2 Hz, 1H), 3.04 (s, 6H), 2.99 (q, *J* = 7.2 Hz, 1H), 2.85 (dt, *J* = 13.1, 6.4 Hz, 1H), 2.75 (d, *J* = 19.1 Hz, 1H), 2.52 (dd, *J* = 14.0, 7.1 Hz, 1H), 2.15 (dd, *J* = 14.0, 7.3 Hz, 1H), 0.84 (s, 9H). ^13^C{^1^H} NMR (176 MHz, CDCl_3_) δ [ppm] = 178.3, 174.1, 168.2, 165.7, 154.3, 144.0, 143.6, 132.2, 129.3, 121.2, 115.7, 115.4, 113.5, 111.0, 88.4, 87.7, 83.5, 70.3, 62.6, 59.3, 43.0, 41.3, 40.2, 37.2, 36.3, 31.6, 26.1. HRMS (ESI) m/z: [M + Na]^+^ Calcd for C_32_H_36_N_2_O_10_Na^+^ 631.2262; found 631.2262.

**(3a*S*,5a*S*,8*R*,9*R*,10a*S*)-9-(*tert*-butyl)-6-(3,4-dihydroxyphenethyl)-9-hydroxy-2,4,7-trioxooctahydro-4*H*,9*H*-furo[3'',2'':2',3']cyclopenta[1',2':3,4]furo[2,3-*b*]pyrrol-8-yl 3-fluorobenzoate** **(BB60)**

Using **method C** with **BB42** (79.9 mg, 0.14 mmol, 1.0 equiv.); yield 17% (23.8 µmol, 13.9 mg); white powder. Purification by column chromatography (SiO_2_, DCM/EtOAc= 5:1→4:1→2:1, v/v).

R*_f_* = 0.72 (DCM/EtOAc = 1:1). ^1^H NMR (700 MHz, Methonal-d_4_) δ [ppm] = 7.65 – 7.63 (m, 1H), 7.60 (d, *J* = 3.1 Hz, 2H), 7.40 (t, *J* = 2.5 Hz, 1H), 6.67 (d, *J* = 3.9 Hz, 2H), 6.54 (d, *J* = 8.0 Hz, 1H), 5.92 (s, 1H), 5.71 (s, 1H), 5.05 (t, *J* = 7.2 Hz, 1H), 3.91 (dt, *J* = 13.8, 6.6 Hz, 1H), 3.48 – 3.44 (m, 1H), 2.96 (d, *J* = 19.4 Hz, 1H), 2.84 – 2.81 (m, 2H), 2.66 (d, *J* = 19.4 Hz, 1H), 2.61 (dd, *J* = 13.9, 7.1 Hz, 1H), 1.93 (dd, *J* = 14.0, 7.3 Hz, 1H), 0.83 (s, 9H). ^13^C{^1^H} NMR (176 MHz, Methonal-d_4_) δ [ppm] = 179.4, 175.8,169.2, 165.6, 163.5, 146.8, 145.4, 132.0, 131.7, 130.6, 127.0, 122.4, 121.1, 117.6, 116.8, 90.0, 87.6, 85.4, 72.8, 64.5, 60.7, 43.2, 43.1, 38.4, 37.4, 33.4, 27.0. ^19^F{^1^H} NMR (471 MHz, MeOD) δ [ppm] = -113.66. HRMS (ESI) m/z: [M + Na]^+^ Calcd for C_30_H_30_FNO_10_Na^+^ 606.1746; found 606.1748.

**(3aS,5aS,8R,9R,10aS)-9-(tert-butyl)-6-(3,4-dihydroxyphenethyl)-9-hydroxy-2,4,7-trioxooctahydro-4H,9H-furo[3'',2'':2',3']cyclopenta[1',2':3,4]furo[2,3-b]pyrrol-8-yl 2-fluorobenzoate** **(BB63)**

Using **method C** with **BB41** (79.9 mg, 0.14 mmol, 1.0 equiv.); yield 31% (43.4 µmol, 25.3 mg); white powder. Purification by column chromatography (SiO_2_, DCM/EtOAc= 4:1, v/v).

R*_f_* = 0.60 (DCM/EtOAc = 1:1). ^1^H NMR (700 MHz, Methonal-d_4_) δ [ppm] = 7.78 (td, *J* = 7.5, 1.8 Hz, 1H), 7.66 (dddd, *J* = 8.7, 7.0, 5.0, 1.8 Hz, 1H), 7.29 (td, *J* = 7.6, 1.0 Hz, 1H), 7.26 – 7.23 (m, 1H), 6.59 (dd, *J* = 8.0, 2.1 Hz, 1H), 6.16 (s, 1H), 5.64 (s, 1H), 5.06 (t, *J* = 7.2 Hz, 1H), 3.83 (dt, *J* = 14.3, 6.2 Hz, 1H), 3.52 (ddd, *J* = 14.4, 8.5, 6.3 Hz, 1H), 2.89 (ddd, *J* = 14.8, 8.5, 6.5 Hz, 1H), 2.85 – 2.82 (m, 1H), 2.82 – 2.76 (m, 2H), 2.63 (dd, *J* = 13.8, 7.2 Hz, 1H), 2.12 (dd, *J* = 13.8, 7.3 Hz, 1H), 0.94 (s, 9H). ^13^C{^1^H} NMR (176 MHz, Methonal-d_4_) δ [ppm] = 179.5, 175.5, 169.0, 163.9, 162.1, 146.7, 145.3, 136.7, 132.6, 130.5, 125.6, 120.9, 118.0, 116.8, 89.9, 87.4, 85.4, 72.5, 64.4, 60.6, 42.9, 42.8, 38.3, 37.2, 33.2, 27.0. ^19^F{^1^H} NMR (471 MHz, MeOD) δ [ppm] = -111.28. HRMS (ESI) m/z: [M + Na]^+^ Calcd for C_30_H_30_FNO_10_Na^+^ 606.1746; found 606.1747.

**(3a*S*,5a*S*,8*R*,9*R*,10a*S*)-9-(*tert*-butyl)-6-(3,4-dihydroxyphenethyl)-9-hydroxy-2,4,7-trioxooctahydro-4*H*,9*H*-furo[3'',2'':2',3']cyclopenta[1',2':3,4]furo[2,3-*b*]pyrrol-8-yl 3-(trifluoromethyl)benzoate** **(BB64)**

Using **method C** with **BB40** (93.9 mg, 0.14 mmol, 1.0 equiv.); yield 29% (40.6 µmol, 25.7 mg); white powder. Purification by column chromatography (SiO_2_, DCM/EtOAc= 5:1→4:1→3:1, v/v).

R*_f_* = 0.65 (DCM/EtOAc = 1:1). ^1^H NMR (700 MHz, Methonal-d_4_) δ [ppm] = 8.24 (s, 1H), 7.97 (t, *J* = 8.5 Hz, 2H), 7.82 (t, *J* = 7.7 Hz, 1H), 6.69 – 6.64 (m, 2H), 6.55 (dd, *J* = 8.2, 2.1 Hz, 1H), 5.94 (s, 1H), 5.74 (s, 1H), 5.05 (t, *J* = 7.1 Hz, 1H), 3.92 (dt, *J* = 13.8, 6.8 Hz, 1H), 3.45 (dt, *J* = 13.6, 6.4 Hz, 1H), 2.96 (d, *J* = 19.4 Hz, 1H), 2.84 (t, *J* = 6.8 Hz, 2H), 2.66 (d, *J* = 19.4 Hz, 1H), 2.61 (dd, *J* = 14.0, 7.1 Hz, 1H), 1.95 (dd, *J* = 13.9, 7.3 Hz, 1H), 1.13 (s, 1H), 0.85 (s, 10H). ^13^C{^1^H} NMR (176 MHz, Methonal-d_4_) δ [ppm] = 178.8, 174.3, 169.7, 165.5, 146.7, 145.3, 131.0, 130.3, 121.0, 116.7, 116.6, 92.7, 90.9, 85.6, 72.7, 62.9, 61.7, 42.7, 42.2, 38.3, 36.1, 32.9, 27.8. ^19^F{^1^H} NMR (471 MHz, MeOD) δ [ppm] = -64.36. HRMS (ESI) m/z: [M + Na]^+^ Calcd for C_31_H_30_F_3_NO_10_Na^+^ 656.1714; found 656.1711.

**(3a*S*,5a*S*,8*R*,9*R*,10a*S*)-9-(*tert*-butyl)-6-(3,4-dihydroxyphenethyl)-9-hydroxy-2,4,7-trioxooctahydro-4*H*,9*H*-furo[3'',2'':2',3']cyclopenta[1',2':3,4]furo[2,3-*b*]pyrrol-8-yl 4-(trifluoromethyl)benzoate (BB65)**

Using **method C** with **BB39** (93.9 mg, 0.14 mmol, 1.0 equiv.); yield 23% (32.2 µmol, 20.4 mg); white powder. Purification by column chromatography (SiO_2_, DCM/EtOAc= 4:1→3:1→2:1, v/v).

R*_f_* = 0.54 (DCM/EtOAc = 1:1). ^1^H NMR (700 MHz, Methonal-d_4_) δ [ppm] = 8.13 (d, *J* = 8.2 Hz, 2H), 7.83 (d, *J* = 8.2 Hz, 2H), 6.72 (d, *J* = 8.0 Hz, 1H), 6.70 (d, *J* = 2.1 Hz, 1H), 6.59 (dd, *J* = 8.0, 2.1 Hz, 1H), 6.21 (s, 1H), 5.66 (s, 1H), 5.12 (t, *J* = 7.1 Hz, 1H), 3.83 (dt, *J* = 14.1, 6.1 Hz, 1H), 3.56 – 3.50 (m, 1H), 2.92 – 2.87 (m, 2H), 2.85 – 2.81 (m, 2H), 2.68 (dd, *J* = 13.9, 7.2 Hz, 1H), 2.07 – 2.04 (m, 1H), 0.92 (s, 9H). ^13^C{^1^H} NMR (176 MHz, Methonal-d_4_) δ [ppm] = 179.2, 175.9, 168.9, 165.5, 146.7, 145.3, 131.6, 130.5, 129.0, 126.8, 121.0, 116.9, 116.7, 89.8, 87.4, 85.4, 72.9, 64.4, 60.7, 43.2, 38.3, 37.3, 35.9, 33.3, 26.9. ^19^F{^1^H} NMR (471 MHz, MeOD) δ [ppm] = -64.81. HRMS (ESI) m/z: [M + Na]^+^ Calcd for C_31_H_30_F_3_NO_10_Na^+^ 656.1714; found 656.1712.

### 2.1.5. The synthesis of **BB66** ~ **BB68**

**(2*R*,3a'*S*,4*R*,6'*R*,7a'*S*)-6'-(*tert*-butyl)-2',4',5-trioxohexahydro-4'*H*,6'*H*-spiro[furan-3,8'-[3a,6]methanofuro[3,2-*c*]pyran]-2,4-diyl bis(4-(((tert-butoxycarbonyl)amino)methyl) benzoate) (BB66)**

4-(((tert-butoxycarbonyl)amino)methyl)benzoic acid (**M-23**, 1.51 g, 6.0 mmol, 4.0 equiv) was dissolved in dichloromethane (15 mL) and Oxalyl chloride (512 µL, 6.0 mmol, 4.0 equiv) was added at 0 ℃. Then one drop of N,N-Dimethylformamide was added. The mixture was stirred for 30 min. By TLC, the endpoint of the reaction was detected. The Bilobalide (489 mg, 1.5 mmol, 1.0 equiv) followed by TMEDA (1.754 mL, 12.0 mmol, 8.0 equiv) was added. The reaction was stirred at room temperature for 20 h. With the reaction finished, the solution was concentrated under reduced pressure, diluted with saturated NaHCO_3_. The reaction solution was extracted with DCM (45 mL), sequentially washed with water and saturated NaCl solution. The organic phase was dried over anhydrous MgSO_4_, filtered, and concentrated under reduced pressure. The residue was purified by column chromatography to give compound **BB66** (1000 mg, 84% yield, white powder).

R*_f_* = 0.35 (hexane/EtOAc = 1:1). ^1^H NMR (700 MHz, CDCl_3_) δ [ppm] = 8.01 (d, *J* = 7.9 Hz, 2H), 7.96 (d, *J* = 7.8 Hz, 2H), 7.47 (d, *J* = 7.7 Hz, 2H), 7.41 (d, *J* = 7.7 Hz, 2H), 7.33 (s, 1H), 6.21 (s, 1H), 5.15 (s, 1H), 5.05 (s, 1H), 4.46 (s, 1H), 4.44 (s, 2H), 4.40 (s, 2H), 3.32 (d, *J* = 17.9 Hz, 1H), 3.09 (d, *J* = 17.9 Hz, 1H), 2.85 (dd, *J* = 15.2, 4.2 Hz, 1H), 2.81 (dd, *J* = 15.3, 7.7 Hz, 1H), 1.49 (s, 9H), 1.47 (s, 9H), 1.26 (s, 9H). ^13^C{^1^H} NMR (176 MHz, CDCl_3_) δ [ppm] = 173.2, 169.1, 168.9, 163.5, 156.1, 146.9, 146.2, 130.6, 130.5, 127.9, 127.7, 127.0, 126.5, 100.9, 94.3, 80.2, 80.0, 78.6, 67.5, 63.9, 62.7, 60.6, 44.4, 35.8, 34.8, 32.4, 28.5, 27.0, 26.5. HRMS (ESI) m/z: [M + Na]^+^ Calcd for C_41_H_48_N_2_O_14_Na^+^ 815.2998; found 815.2996.

Compound **BB67** was obtained using the synthetic route described for preparation of compound **BB10**.

**(3a*S*,5a*S*,8*R*,9*R*,10a*S*)-9-(*tert*-butyl)-6-(3,4-dihydroxyphenethyl)-9-hydroxy-2,4,7-trioxooctahydro-4*H*,9*H*-furo[3'',2'':2',3']cyclopenta[1',2':3,4]furo[2,3-*b*]pyrrol-8-yl 4-(((tert-butoxycarbonyl)amino)methyl)benzoate (BB67)**

Using **method C** with **BB66** (200 mg, 0.25 mmol, 1.0 equiv.), **M-22** (52.1 mg, 275 µmol, 1.1 equiv.), TEA (488 µL, 3.50 mmol, 14 equiv.) and ACN (2.5 mL); yield 67% (168 µmol, 116 mg); white powder. Purification by column chromatography (SiO_2_, DCM/EtOAc= 1:1, v/v).

R*_f_* = 0.60 (DCM/EtOAc = 1:1). ^1^H NMR (700 MHz, CDCl_3_) δ [ppm] = 8.06 – 8.01 (m, 1H), 7.90 (d, *J* = 8.1 Hz, 2H), 7.37 (dd, *J* = 8.4, 3.8 Hz, 2H), 6.79 (d, *J* = 8.1 Hz, 1H), 6.72 (d, *J* = 2.0 Hz, 1H), 6.62 (dd, *J* = 8.1, 2.0 Hz, 1H), 6.05 (s, 1H), 5.55 (s, 1H), 5.14 (t, *J* = 7.1 Hz, 1H), 4.39 (d, *J* = 6.2 Hz, 1H), 4.37 (t, *J* = 5.0 Hz, 1H), 3.90 (dt, *J* = 13.4, 6.4 Hz, 1H), 3.50 (dt, *J* = 11.7, 7.1 Hz, 1H), 2.96 (d, *J* = 19.4 Hz, 2H), 2.86 (dt, *J* = 14.6, 6.3 Hz, 1H), 2.73 (d, *J* = 19.1 Hz, 1H), 2.55 (dd, *J* = 14.1, 7.2 Hz, 1H), 2.12 (dd, *J* = 14.1, 7.0 Hz, 1H), 1.46 (s, 9H), 0.86 (s, 9H). ^13^C{^1^H} NMR (176 MHz, CDCl_3_) δ [ppm] = 178.0, 173.9, 169.0, 167.6, 165.4, 156.2, 146.1, 144.1, 143.5, 130.6, 129.3, 127.5, 126.6, 121.1, 115.7, 115.4, 88.3, 87.5, 83.4, 80.2, 70.9, 62.6, 59.4, 44.3, 43.1, 41.5, 37.2, 36.3, 31.8, 28.5, 26.2. HRMS (ESI) m/z: [M + Na]^+^ Calcd for C_36_H_42_N_2_O_12_Na^+^ 717.2630; found 717.2631.

(4-**((((3a*S*,5a*S*,8*R*,9*R*,10a*S*)-9-(*tert*-butyl)-6-(3,4-dihydroxyphenethyl)-9-hydroxy-2,4,7-trioxooctahydro-4*H*,9*H*-furo[3'',2'':2',3']cyclopenta[1',2':3,4]furo[2,3-*b*]pyrrol-8-yl)oxy)carbonyl)phenyl)methanaminium chloride** **(BB68)**

Methanol (6 mL) was cooled to 0 ℃ and acetyl chloride (228 µL, 3.2 mmol, 20.0 equiv) was added, resulting in a 0.5 M HCl solution. Then **BB67** (111 mg, 0.16 mmol, 1.0 equiv) was added and the mixture was stirred at 0 ℃ for 4 h. After the reaction was finished, the solid was obtained with evaporation to produce **BB68** (95.9 mg,152 µmol, 95% yield, white powder).

R*_f_* = 0.026 (DCM/EtOAc = 1:1).^1^H NMR (700 MHz, Methanol-d_4_) δ [ppm] = 8.02 (d, *J* = 8.2 Hz, 2H), 7.60 (d, *J* = 8.3 Hz, 2H), 6.72 (d, *J* = 8.0 Hz, 1H), 6.70 (d, *J* = 2.1 Hz, 1H), 6.58 (dd, *J* = 8.1, 2.1 Hz, 1H), 6.18 (s, 1H), 5.66 (s, 1H), 5.12 (t, *J* = 7.1 Hz, 1H), 4.22 (s, 2H), 3.82 (dt, *J* = 14.2, 6.1 Hz, 1H), 3.53 (ddd, *J* = 14.5, 8.5, 6.3 Hz, 1H), 2.90 – 2.87 (m, 1H), 2.85 (d, *J* = 14.2 Hz, 2H), 2.82 (td, *J* = 7.9, 5.9 Hz, 1H), 2.67 (dd, *J* = 13.8, 7.1 Hz, 1H), 2.02 (dd, *J* = 13.7, 7.1 Hz, 1H), 0.91 (s, 9H). ^13^C{^1^H} NMR (176 MHz, Methanol-d_4_) δ [ppm] = 179.3, 175.9, 169.1, 165.9, 146.7, 145.3, 140.7, 131.6, 130.5, 130.3, 130.1, 121.0, 116.9, 116.7, 89.9, 87.4, 85.4, 72.6, 64.4, 60.6, 43.7, 43.1, 43.0, 38.2, 37.3, 33.3, 26.9. HRMS (ESI) m/z: [M + H]^+^ Calcd for C_31_H_34_N_2_O_10_ HCl^+^, 595.2286; found 595.2286.

## 2.2. Copies of NMR Spectra

**NMR Spectrum of BB37**

**NMR Spectrum of BB38**

**NMR Spectrum of BB39**

**NMR Spectrum of BB40**

**NMR Spectrum of BB41**

**NMR Spectrum of BB42**

**NMR Spectrum of BB43**

**NMR Spectrum of BB44**

**NMR Spectrum of BB45**

**NMR Spectrum of BB46**

**NMR Spectrum of BB47**

**NMR Spectrum of BB48**

**NMR Spectrum of BB49**

**NMR Spectrum of BB50**

**NMR Spectrum of BB51**

**NMR Spectrum of BB52**

**NMR Spectrum of BB53**

**NMR Spectrum of BB54**

**NMR Spectrum of BB55**

**NMR Spectrum of BB56**

**NMR Spectrum of BB57**

**NMR Spectrum of BB58**

**NMR Spectrum of BB59**

**NMR Spectrum of BB60**

**NMR Spectrum of BB63**

**NMR Spectrum of BB64**

**NMR Spectrum of BB65**

**NMR Spectrum of BB66**

**NMR Spectrum of BB67**

**NMR (700MHz, Methanol-d_4_) Spectrum of BB68**
